# Dose-Dependent Onset of Regenerative Program in Neutron Irradiated Mouse Skin

**DOI:** 10.1371/journal.pone.0019242

**Published:** 2011-04-27

**Authors:** Emiliano Fratini, Valerio Licursi, Mara Artibani, Katarzyna Kobos, Paolo Colautti, Rodolfo Negri, Roberto Amendola

**Affiliations:** 1 ENEA, Agenzia nazionale per le nuove tecnologie, l′energia e lo sviluppo economico sostenibile Roma, Italy; 2 Sapienza Università di Roma, Dipartimento di Biologia e Biotecnologie “Charles Darwin”, Roma, Italy; 3 INFN, Laboratori Nazionali di Legnaro, Legnaro, Italy; 4 Istituto Pasteur-Fondazione Cenci Bolognetti, Roma, Italy; French National Centre for Scientific Research, France

## Abstract

**Background:**

Tissue response to irradiation is not easily recapitulated by cell culture studies. The objective of this investigation was to characterize, the transcriptional response and the onset of regenerative processes in mouse skin irradiated with different doses of fast neutrons.

**Methodology/Principal Findings:**

To monitor general response to irradiation and individual animal to animal variation, we performed gene and protein expression analysis with both pooled and individual mouse samples. A high-throughput gene expression analysis, by DNA oligonucleotide microarray was done with three months old C57Bl/6 mice irradiated with 0.2 and 1 Gy of mono-energetic 14 MeV neutron compared to sham irradiated controls. The results on 440 irradiation modulated genes, partially validated by quantitative real time RT-PCR, showed a dose-dependent up-regulation of a sub-class of keratin and keratin associated proteins, and members of the S100 family of Ca^2+^-binding proteins. Immunohistochemistry confirmed mRNA expression data enabled mapping of protein expression. Interestingly, proteins up-regulated in thickening epidermis: keratin 6 and S100A8 showed the most significant up-regulation and the least mouse-to-mouse variation following 0.2 Gy irradiation, in a concerted effort toward skin tissue regeneration. Conversely, mice irradiated at 1 Gy showed most evidence of apoptosis (Caspase-3 and TUNEL staining) and most 8-oxo-G accumulation at 24 h post-irradiation. Moreover, no cell proliferation accompanied 1 Gy exposure as shown by Ki67 immunohistochemistry.

**Conclusions/Significance:**

The dose-dependent differential gene expression at the tissue level following *in vivo* exposure to neutron radiation is reminiscent of the onset of re-epithelialization and wound healing and depends on the proportion of cells carrying multiple chromosomal lesions in the entire tissue. Thus, this study presents *in vivo* evidence of a skin regenerative program exerted independently from DNA repair-associated pathways.

## Introduction

Treatment of radiation injuries still represents an essential challenge based on the uncertainty of many aspects of the pathophysiology of the biological effects of the radiation. Despite the knowledge that ionizing radiation effectively causes DNA damage, numerous reports suggest the existence of complex intracellular and inter-cellular damage signaling pathways in cells, tissues and organs which exert important biological effects [1], [2]. Thus, the energy deposition from ionizing radiation should be not to be considered only at the level of the DNA but also at the level of cell populations. Especially research on low dose and/or not uniform irradiation exposures often enables one to observe these effects [3], [4]. High linear energy transfer radiation (high-LET) represents an ubiquitous and potentially dangerous hazard even when associated with the use of radiation as diagnostic tools [5], [6], and at low doses of environmental cosmic rays, during low earth astronautic orbit mission and aircrew activity [7], [8]. Indeed, epidemiological evidence from human populations demonstrates that high LET doses above 50–100 mSv for protracted exposure, or 10–50 mSv for acute exposure, increases the risk of cancers [1], [9], [10]. While these facts already justify the efforts to gain knowledge on biological effects of high LET radiation there is also a possibility for the therapeutic use of this type of radiation [11]. High-LET radiation is relatively more effective against tumors than low LET because it causes cellular damage almost independently of cellular redox metabolism and proliferation and produces more complex lesions than those created by sparsely ionising radiations such as low-LET radiations [12], [13]. As a result, DNA damage from high LET radiation is most often by large deletions and rarely corrected [14].

Thus, the assessment of the potential role for high-LET radiation in the treatment of cancer is important as well as determining the tolerance of the contiguous normal tissues to these exposure [15], [16]. Regardless of the cause of high LET exposure (accidental or radiotherapy) the skin's reaction to irradiation is a key diagnostic and prognostic factor to take into account [16], [17]. Cutaneous radiation syndrome, depending on the quality and quantity of the radiation dose, involves every single component of the cell's life, including alterations in cell–to-cell crosstalk and cell–matrix interaction. In the early phases after radiation exposure, the cell produces some cytokines (*IL (interleukin)-1*, *IL*-*6*, tumor necrosis factor *(TNF)-α*, transforming growth factor *(TGF)-ß*, the chemokines *IL-8* and eotaxin) [18] and an inter-cellular adhesion molecule-1 (*ICAM-1*) [19] as parts of the tissue's response to injury. These activities of skin's cells suggest evidence of molecular signaling radiation responses which may not be directly linked to DNA damage. However, knowledge of the role of cytokines and inflammation in radiation response is not yet clear [20]. While fractionated irradiation may prevent skin inflammation iniatially, unacceptable latent effects can occur years later [21]. Finally, clinical observations of normal tissue damage following radiotherapy support the idea that genetic differences among patients account for much of an individual's unpredictable response to either quality of radiation [22]-[24].

Recently, high-throughput gene expression analyses have been performed to identify patterns of molecular changes following exposure of the skin to irradiation. The goal of these studies was to establish a panel of radiation responsive genes and discover new molecular biological markers of radiation response [25]. However, these studies were principally done with fibroblasts [26], [27] or keratinocytes cell lines [28], [29], and the most consistent results concerned p53 [25], [30] and NF-kB [30], [31] responsive genes since DNA damage and inflammation are common negative outcomes of radiation exposure.

The objective of this research, was to gain insight into the individual response of gene expression, intracellular and inter-cellular signaling pathways and membrane-mediated signaling at the cell and tissue level in the skin of mice treated by mono-energetic 14 MeV neutron irradiation. Skin irradiation schemes were based on the cumulative probability to have different proportion of cells carrying multiple lesions post-irradiation if the target site size is around 1 µm. This probability was twenty percent lower for the skin of mice exposed to 0.2 Gy than those exposed to 1 Gy dose, which is the daily dose used in protocols of fractionated neutron therapy [32]–[34].

As mentioned before, high-throughput gene expression analyses have frequently been used to identify patterns of molecular changes that occur following exposure. A resultant panel of radiation responsive genes can be useful for the followup of radiation therapy, and even for triage of individuals in accidentally exposed populations [35]. Most of the latest evidence on skin reaction after ionizing radiation *in-vivo is* focused on inflammation [18], [19] and DNA damage repair [36]. *In-vitro* experiments with low-LET irradiation, for example, addressed specifically the crucial role of *p53* after exposure of the human HaCaT keratinocyte cell line [28]. Nevertheless, three-dimensional skin architecture is of fundamental importance for stress response [37], [38] and the expression of *p53* expression fails to be a predictive indicator of pathological outcomes of skin in patients [24]. At the same time, in many *in vivo* studies, especially with relatively low doses of radiation, individual animal-to-animal variation proved to have overwhelming effects sometimes obscuring more universal effects of treatments. The aim of this study was to compile a comprehensive *in-vivo* study, which would span across interrogation of (i) universal skin tissue changes in gene expression in pooled RNA samples; (ii) skin tissue changes in specific mRNA expressions in individual mice; and (iii) animal specific and cell specific changes in protein expression as monitored by immunohistochemistry. With this study design we hoped to address both universal gene expression changes for a skin wide complex response to high LET, as well as cell specific and cell type specific gene expression response to 14 MeV neutrons, in keeping with individual variation. We found a co-regulation of a sub-class of keratin, keratin associated protein and S100 family of Ca^2+^-binding proteins which were up-regulated after the 0.2 Gy dose irradiation, and that were not modulated or down-regulated after the dose of 1 Gy. The differential modulation of these genes in the epidermis can be partially associated to the onset of re-epithelialization processes [39], and characterized by a substantial lack of apoptosis or accumulation of oxidized DNA observed at 24 hours after 0.2 Gy exposure, opposite from situation in mice exposed to 1 Gy. These repair processes recorded for individual mice are more likely to be associated with *in-vivo* re-modeling of skin architecture than with DNA repair processes.

## Materials and Methods

### Irradiation of experimental animals

The animal's welfare is checked regularly, and the ENEA Casaccia Research Center's animal facility complies with the national and international laws and regulations. To comply with the Italian Ministry of Health art. 3, 4, 5 of law number 116/92, the experiment plan was submitted to the ENEA Local Ethics Committee for Animal Research (www.bologna.enea.it/matform/Biotec/Tirindelli2.pdf *)*, for the necessary advance authorization. The ENEA Local Ethics Committee for Animal Research established absolute absence of any stress and pain for the treated mice and, in compliance with the above cited Italian laws and regulations, the Italian Ministry of Health's approval was not necessary. Three-months-old C57Bl/6 *mus musculus* male mice (Charles River, Como, Italy) were hosted at the animal house, and fed with commercial pellet and chlorinated water *ad libitum*. The animal room was maintained at 20–25°C and 55–65% humidity with a 12 h light dark cycle. After ten days of acclimatization, the mice were randomly assigned to different experimental groups. Each experimental group included at least ten mice. The mice, restrained in plastic boxes, received total body irradiation with 0, 0.2 and 1 Gy of 14 MeV neutron doses, generated by the linear electrostatic accelerator Frascati Neutron Generator (FNG, ENEA, Frascati, Italy) via the Deuterium-Tritium (D+T) reaction [40]. The dose rate was 25 cGy/min for the 1 Gy exposure, and 6 cGy/min for the 0.2 Gy exposure, to minimize any difference due to timing of treatments, and considering previous experimental evidences of negligible dose rate effects compared to total effects following *in vivo* neutron irradiation [41], [42]. At six and at twenty-four hours after exposure, the mice were lethally anesthetized with Avertin-R, (1∶1 w/v solution of 2,2,2-tribromoethanol, tert-amyl alcohol, in 39 ml of H_2_O), administered intra-peritoneally [43]. Mouse skin dissection was identically performed from each mouse. Skin was shaved by three passages of dedicated electrical razor and one sample of dorsal skin, comprised of both epidermis and dermis, was excised. One piece was fresh frozen in liquid nitrogen and keep at −80°C until RNA isolation, and one piece was paraffin embedded.

### Neutron Microdosimetric calculations

High-LET radiations differ from low-LET radiations because of the larger ionization induced by a single ionization event in nanometric sites. In micrometric sites instead, the ionization can be increased also by multiple ionization event numbers ***n***, which increase with the absorbed dose ***D***. Therefore, we have calculated the multiple ionization event numbers (***n*** *)* for different site sizes and the absorbed dose (***D*** *)* values by using microdosimetric algorithms (Appendix S1). For calculations we have taken into account only 14 MeV neutrons dose delivered, since gamma dose contamination during a 14 MeV neutron irradiation comes from very few 16.7 MeV gamma rays via D+T→γ +^5^He (the branching ratio is about 5·10^−5^ [44]), and from neutron-induced nuclear reactions occurring both in the irradiation setup and in the irradiated mouse. The measured less than 4% of gamma dose contamination during a 14 MeV neutron irradiation was in agreement with previous measurements [45]. Therefore, assuming that our radiobiological effects were mainly due to neutrons, we have calculated how much the probability ***F_2_*** to have more than one ionization event in the site increases, when the dose increases from 0.2 to 1 Gy (Fig. S2). In order to calculate ***F_2_***_**,**_ microdosimetric spectra have been generated from secondary particle equilibrium spectra, due to mono energetic 14 MeV neutrons by using the Caswell and Coyne model [46] ***F_2_*** has been calculated also by using the mean microdosimetric value ***y_F_***, which has been measured by Srdoč and Marino [45] in a 14 MeV neutron field after having subtracted the gamma component of the dose (Appendix S1 and Fig. S2).

### RNA extraction and quality analysis

Total RNA was isolated using TriPure Isolation Reagent from Roche Applied Science (Roche, Indianapolis, IN). RNA concentration and purity were determined by measuring absorbance using NanoDrop 1000 Spectrophotometer (Thermo Scientific, Wilmington, DE); 1 µg of total RNA was run on a 1% denaturing gel to verify RNA integrity.

### RNA and Microarray Probe Preparation and Hybridization

Aliquots of 163 ng of total RNA from each experimental mouse belonging to each experimental group were pooled and amplified using the Ambion Amino Allyl MessageAmp™ II aRNA Amplification Kit (AM1753, Applied Biosystems/Ambion, Austin, TX). Amino allyl UTP is incorporated during the transcription step to produce amino allyl modified amplified RNA (aRNA). The aRNA was ready for coupling to the NHS ester label (Cy3 and Cy5) (GE Healthcare, Milano, Ialy, EU). All the procedures described above were repeated to verify experimental reproducibility. The micro-arrays used in this study contained 28000 exons spotted as 70-mer oligonucleotides from the Array-Ready Oligo Set for the mouse genome v3.0 (OPERON, Micro-array Facility Service of the Microarray Consortium of Norwegian University of Science and Technology, Norway, EU). Slides were pre-hybridized at 42°C for at least 45 min in a solution containing 5×SSC, 0.1% SDS and 0.1% BSA. The labeled aRNAs (Cy3 sample and Cy5 sample mixed) were added to 50 µl of hybridization buffer containing 50% formamide, 10× SSC, 0.2% SDS pre-heated at 95°C for 3 min. Hybridization was carried out for 16 h at 42°C and unbound DNA was washed off using 3 steps with solutions containing: I. 1×SSC 0.2% SDS pre-heated at 42°C; II. 0.1×SSC 0.2% SDS; III. two times 0.1×SSC. A PerkinElmer ScanArray Lite Micro-array Scanner was used to acquire images, and GenePix Pro 6.1 software and ScanArray Express software were used to quantify hybridization signals (PerkinElmer, Waltham, MA). Absent and marginal spots were flagged automatically by software and subsequently each slide was inspected individually. We filtered the data to exclude artifacts, saturated spots, and low signal spots. Assuming that most of the genes had not changed expression, the Cy5/Cy3 ratios were normalized using Goulphar script [47] running on open source R software (http://www.r-project.org/), using a Global Lowess Normalization. The hierarchical gene-clustering analyses were performed by TIGR MeV (MultiExperiment ViewerVersion) 4.0 [48]. The parameters used for the hierarchical clustering were the Euclidean distance and the average linkage method. A functional Annotation Clustering report of 440 genes modulated at least 1.5 fold in two different conditions in comparison to the sham-irradiated control using DAVID (http://david.abcc.ncifcrf.gov/home.jsp) web tool [49] is shown in appendixes material Table S1. The complete microarray dataset is MIAME compliant and the raw data has been deposited in the MIAME compliant database Gene Expression Omnibus (GEO) with the accession number GSE25343.

### Real time qPCR (RT-qPCR)

1 µg of total RNA from each individual mouse was retro-transcribed into cDNA by SuperScript First-strand Synthesis System (Invitrogen, Carlsbad, CA), following the manufacturer's instructions. qPCR was performed using the TaqMan Gene Expression Assays system from Applied Biosystems (ABI, Foster City, CA), according to manufacturer's instructions. Amplification included the following *mus musculus* genes: mouse *glyceraldehyde phosphate dehydrogenase* (*GAPDH*) (4352339E, VIC/MGB Probe), *CDKNA1a* (*p21*) (Mm00432448_m1, FAM/MGB Probe), *keratin 10* (*KRT10*) (Mm03009921_ml, FAM/MGB Probe), *keratin 27* (*KRT27*) (Mm00839780_m1, FAM/MGB Probe), *leptin* (Mm00434759_m1, FAM/MGB Probe), Ca^2+^-binding protein *S100A3* (Mm00478587_m1, FAM/MGB Probe). To further support transcriptome data, individual RNA analysis was performed on the additional four mice which were used for immunohistochemistry in parallel. For the following mouse genes, RT-qPCR were performed by SYBR Premix Ex Taq (Takara BIO Inc, Shiga, Jp) according to the manufacturer's instructions: *keratin 6* (*KRT6*), *keratin 14* (*KRT14*), Ca^2+^-binding protein *S100A8*, Ca^2+^-binding protein *S100A9*, *heme-oxygenase1* (*HMOX-1*), and *GAPDH.* Specific primers pairs were designed by Primer Express Software (ABI) and their sequences are listed in Table 1. All reactions were performed in duplicate in the ABI 7300 Real Time PCR System, and relative quantification was carried out with the ΔΔCT method provided by ABI, using the abundance of *GAPDH* mRNA as endogenous house-keeping control.

**Table 1:** Primers for the amplification of genes of interest.

Primers	Sequence	PCR Products Length (bp)
	**5′**-**3′**	
**Krt14 – FOR**	TTCTCCTCTGGCTCTCAGTCATC	**80**
**Krt14 - REV**	TCGTGCACATCCATGACCTT	
**Krt6 - FOR**	TTCTCTACTTCCCAGCCTTCTCA	**80**
**Krt6 - REV**	GCCACGGTGGCTGGTTT	
**S100A8 – FOR**	CCTTGCGATGGTGATAAAAGTG	**82**
**S100A8 - REV**	CCCAGCCCTAGGCCAGAA	
**S100A9 – FOR**	CAAAGGCTGTGGGAAGTAATTAAGA	**72**
**S100A9 - REV**	AAGCCATTCCCTTTAGACTTGGT	
**Hmox-1 – FOR**	GGCAGTGGGAATTTATGCCA	**71**
**Hmox-1 - REV**	GGCCACATTGGACAGAGTTCA	
**Gapdh – FOR**	ATGTGTCCGTCGTGGATCTGA	**81**
**Gapdh – REV**	ATGCCTGCTTCACCACCTTCT	
**FOR – forward**		
**REV – reverse**		

### Dorsal skin immunohistochemistry

Immunohistochemistry analysis was performed for KRT6, KRT10, S100A8, S100A9, Caspase-3, and Ki67 genes, on four additional mice, to search for proof of gene expression modulation at protein level, details of proteins cellular localization and the influence of individual variation on susceptibility to irradiations. Dorsal skin samples were fixed overnight with neutral buffered 4% para-formaldehyde at 4°C and paraffin embedded. Consecutive five-µm sections were cut and mounted onto amino-silane positively charged slides. To retrieve the antigenicity, the tissue sections were twice microwaved (650 W) in 10 mmol/L citrate buffer (pH 6.0). Immunohistochemistry was performed on four randomly chosen slides for each mouse using the peroxidase method (Vectastain Elite ABC kit, Vector Lab., Burlingame, CA) and detected by the NovaRED system (Vector Lab.) following the manufacturer's instructions. A panel of primary antibodies were: rabbit polyclonal anti-KRT6 (MK6, Berkeley Antibody Company, BAbCO, Richmond, CA) rabbit polyclonal anti-KRT10 (Mk10, BabCO), goat polyclonal anti-S100A8 (Calgranulin A, Santa Cruz Biotechnology, Santa Cruz, CA), mouse monoclonal anti-S100A9 (Calgranulin B, Santa Cruz), rabbit polyclonal anti-Caspase 3 (H-277, Santa Cruz), and goat polyclonal anti-Ki67 (M19, Santa Cruz). Primary antibodies were diluted according to the manufacturer's instructions. The anti-mouse and anti-rabbit secondary antibodies were part of customized Vectastain Elite ABC kits. The horseradish peroxidase (HRP) anti-goat secondary antibody was from Santa Cruz. Negative controls were prepared by omitting primary antibodies for all antigens, these data were used to determine background values. Slides were faintly counterstained with hematoxylin to evaluate general skin morphology, and magnified to 400x or phase-contrast 1000x (Ki67) using the Axioskop Zeiss microscope (Zeiss, Oberkochen, Germany) equipped with a DMC2 digital camera (Polaroid, Waltham, MA) and the proprietary image acquisition software. Arbitrary units of optical density were obtained by the image analysis open source software ImageJ (http://rsb.info.nih.gov/ij) without any prior digital modification. Ki67 quantification was obtained by scoring the frequency of positively stained cells. After normalization for the intensity of background staining, at least four areas for each slide were scored for covering of the epidermal layer and hair follicles.

### Dorsal skin evaluation of terminal deoxynucleotidyl transferase Biotin-dUTP nick end labeling (TUNEL) and levels of 8-oxo-7,8-dihydroguanine (8-oxo-G) residues

TUNEL evaluation was performed on four randomly chosen slides for each mouse using In Situ Cell Death Fluorescence kit (Roche) following the manufacturer's instructions. The negative control was obtained by omitting enzyme-mix from the reaction. After normalization for the intensity of background staining, at least four areas measured for each slide were recorded for the epidermal layer and hair follicles. Evaluation of 8-oxo-G residues was performed on four randomly chosen slides for each mouse using fluorimetric OxyDNA assay kit (Merck KGaA, Darmstadt, Germany, EU) following the manufacturer's instructions. The negative control was obtained omitting fluorescence probe-mix from the reaction. Slides were magnified to 400x (objective 40X, 0.75 aperture) using a 50 watt mercury lamp equipped Axioskop Zeiss microscope and digital photographs were taken as described above. After normalization for the intensity of background staining, at least four areas measured for each slide were recorded, covering epidermal layer and hair follicles.

### Statistical analysis

The data were presented as whisker box plots, to show the incidence of data coming from outliers. Significant differences at p<0.05 were evaluated by the one-way ANOVA, followed by the multiple comparison Tukey post-hoc test (SPSS-11 statistical dedicated software - SPSS Inc., Chicago, IL). All experiments were repeated four times unless otherwise indicated.

## Results

### Neutron Microdosimetric calculations

Fig. S2 shows that the probability to have multiple ionization events in the biological site increases with the site diameter and with the absorbed dose. However, if the site diameter is as large as the cell diameter (average size considered as 7 µm), multiple events occur always in the site both at 0.2 Gy and 1 Gy. Similarly, if the site diameter is very small (less than 0.5 µm), hardly multiple events occur in the site both at 0.2 Gy and 1 Gy (the probability is much less than 1%). A significantly different incidence occurs only if the site size is in between 1 µm and 2 µm. In fact, in these sites multi events occurrence increases from 0.3% to 6.5% and from 4.4% to 49% respectively, when the absorbed dose increases from 0.2 Gy to 1 Gy.

### Dose-dependent transcriptome modulation of neutron-irradiated skin

**Figure 1:**
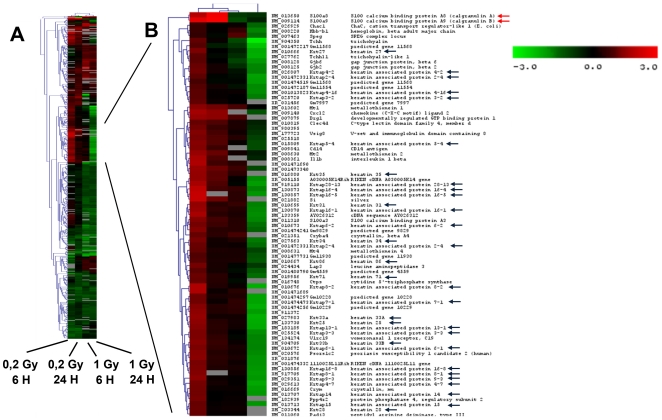
Transcriptome analysis of neutron-irradiated skin. Panel A. Hierarchical cluster (Euclidean distance, average linkage) and heat map of 440 genes modulated more than 1.5 fold in at least two conditions as compared to sham irradiated controls examined as follows: 0.2 Gy at 6 h; 0.2 Gy at 24 h; 1 Gy at 6 h; 1 Gy at 24 h. Signal intensity is represented by color in a log2 scale from -3 to +3, where red is the highest and green is the lowest. Panel B. Zoom-in of a portion of hierarchical clustering. Genes show induction at 6 h at 0.2 Gy and repression or no modulation at 1 Gy 14 MeV neutron dose. This gene cluster is particularly enriched with keratin and keratin-associated genes (black arrows) and contains some S100 Ca^2+^-binding protein genes (red arrows).

**Figure 2:**
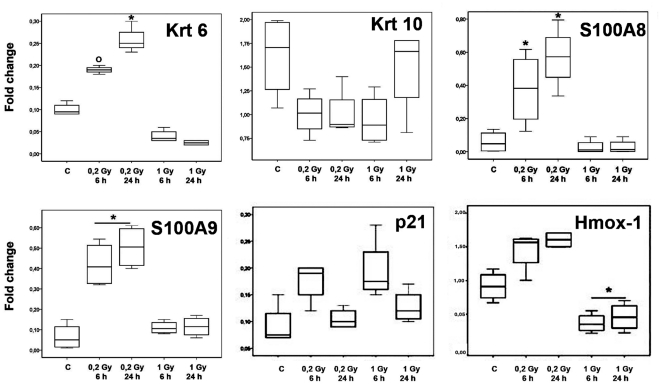
RNA expression of *KRT6, KRT10, S100A8, S100A9, p21, and HMOX-1*. Fold change of RNA expression in dorsal skin samples of individual mice was done on the additional four mice per group and normalized to the housekeeping gene *GADPH* and plotted as whisker boxes as indicated for each gene analyzed. Statistical analysis for *KRT6* showed a level significantly (p<0,05) higher for mice treated with 0.2 Gy at 24 hours (*) and for 0.2 Gy at 6 hours (O) with respect to the sham irradiated and 1 Gy group of mice. *KRT10* showed a tendency to decrease (statistically not significant) in all treated samples. Both the S100A8 and S100A9 genes showed a significantly (p<0,05) higher level for mice treated with 0,2 Gy at 6 and 24 hours (*). Statistical analysis for p21 showed a tendency of this mRNA to increase (statistically not significant) in all treated samples at 6 hours after irradiation, more pronounced at 1 Gy. *HMOX-1* showed a significantly (p<0,05) lower level at both time points after exposure at 1 Gy.

We applied DNA microarrays methodology to analyse transcriptomic changes following skin exposure to different doses of 14 MeV neutron irradiation. We identified genes modulated at least 1.5 fold in two different conditions (5.7% of all examined genes) in comparison to the sham-irradiated control. This list was filtered on the base of consistency in replicated experiments by Student t-tests (P<0.05). The final list contains 440 genes (Table S1). Gene ontology analysis shows that differentially expressed genes are mostly involved in cystoskeleton, extracellular matrix organization and biogenesis, and cell communication, inflammation, stress and immune responses (Table S2). Most of these genes were never previously identified to be modulated by irradiation in cultured keratinocytes [27] and their coordinated changes could reflect an organized tissue response to irradiation. In order to group the modulated genes in function of their responses to the different doses and times of sampling, we applied hierarchical clustering analysis (Fig. 1A). Large clusters which show different modulation at the two doses were evident. We were particularly interested in the behaviour of two sub-clusters respectively containing mRNAs coding for keratins and keratin-associated proteins (Fig. 1A, black arrows), and S100 calcium binding proteins (Fig. 1A, red arrows). A large portion of keratins and keratin-associated proteins, most of which are physically associated in genomic clusters, behaved similarly, showing induction at 6 h following 0.2 Gy irradiation and repression or no modulation at the higher dose. Similar pattern was evident for some of the S100 calcium binding proteins, previously proven to be involved in skin response to damage [50]. It was crucial to validate some of these modulations by an independent technique. Fig. S1 shows that there is a high positive correlation between the average modulations assessed by microarrays and by RT-qPCR (R = 0.88; p-value = 9.7E-14). Moreover, analysis on individual mice, performed on all the genes analyzed by RT-qPCR (10 genes for 4 different conditions, total 40 experimental points per mouse), showed that the observed modulations are not due to outlier effects (Fig. 2). On the whole, these data showed that gene expression at 0.2 Gy is related to an early coordinated activation of a series of genes that manage skin tissue organization and damage response, and are absent after the exposure to 1 Gy. However, transcriptomic data cannot explain if these modulations are caused by a limited response of a large number of cells or bya dramatic change in a small number of cells which command the tissue's response. It was therefore crucial to identify the type of cells involved in these tissues responses and localize some of the putatively radiation-modulated proteins by immunohistochemistry.

### The onset of skin repair after neutron irradiation was dose dependent

**Figure 3:**
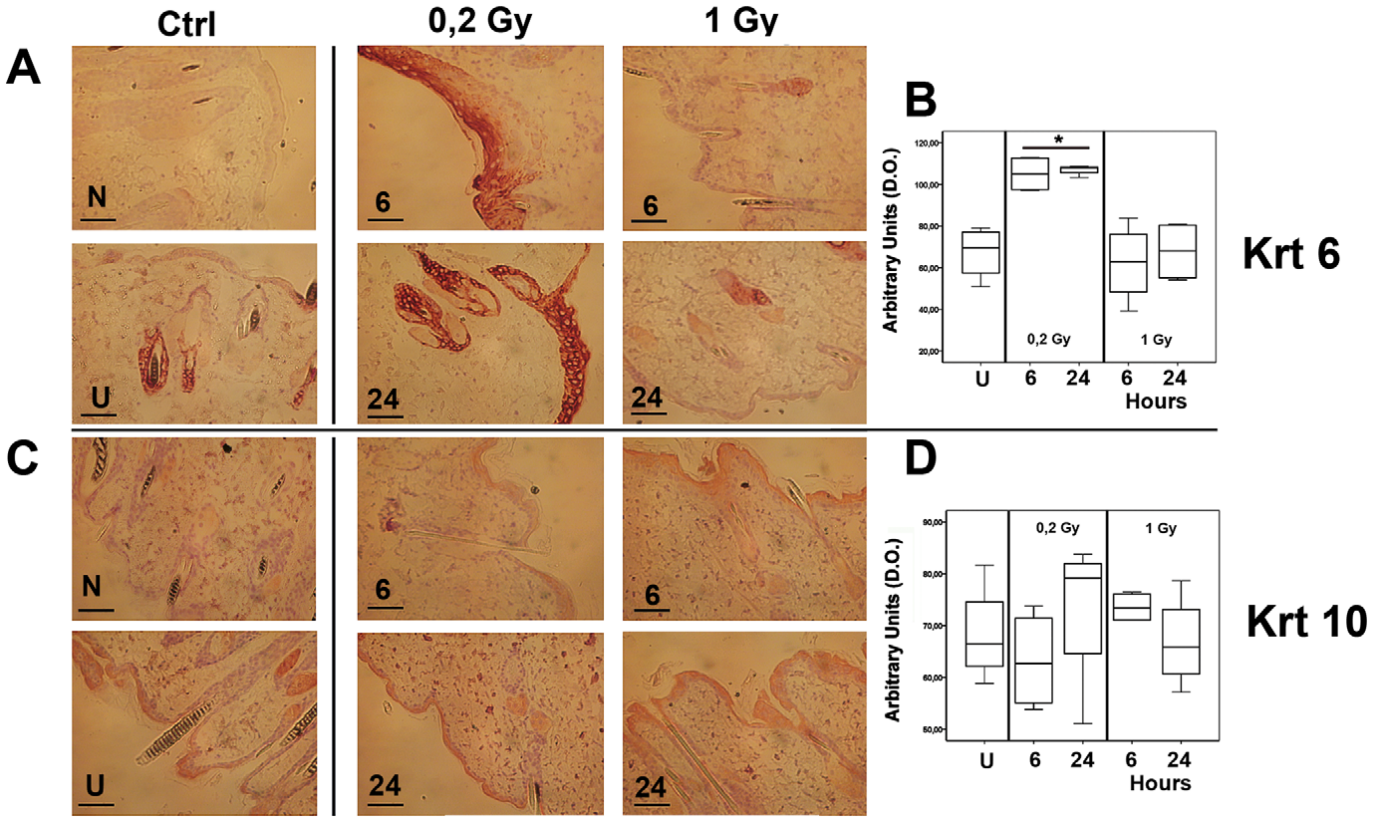
Immunoreactivity for KRT6 and KRT10. Representative images are reported in Panel A for Krt6, and Panel C for Krt10. Scale black bar represents 20 µm. Abbreviations: N = negative controls obtained by omission of the primary antibodies; U = untreated sham irradiated samples; 6, 24 = hours of sampling after irradiation, respectively with 0.2 and 1 Gy of 14 MeV neutron irradiation. Krt6 was cytoplasmic accumulated at the lower dose of 0.2 Gy group (panel A), with a thickening of epidermis as compared with sham and 1 Gy irradiated mice. Arbitrary densitometric units of protein expression are plotted as whisker box in panel B, and showed limited individual variation within the groups. Statistical analysis showed a significantly (p<0,05) higher level for the group of mice treated with 0.2 Gy irradiation (*). KRT10 did not show a statistically significant variation among the experimental mice (Panel C). Arbitrary densitometric units of protein expression are plotted as whisker box in panel D.

As shown in Fig. 3A, KRT6 was accumulated in response to 0.2 Gy, both at 6 and 24 hours after irradiation. Epidermal keratinocytes and hair follicles were uniformly labeled, and a thickening of epidermis was evident as compared with sham and 1 Gy irradiated mice. Arbitrary densitometric units analysis are reported in the form of whisker box plot in Fig. 3B, showing statistical significance with limited individual variation within the groups (Table S3). These results are consistent with an increased RNA expression at 0.2 Gy with narrow range of individual variability, as indicated by the fold change normalized to the housekeeping gene *GAPDH* and depicted in the whisker box plot in Fig. 2. This pattern, coupled with mRNA over expression of *KRT16* and *KRT17* at 0.2 Gy detected by cDNA microarray (Fig. 2 and Table S1), is similar to the multi-faceted skin tissue re-epithelialization program of injured skin, described to be associated with down-regulation of the differentiation-specific keratins KRT10 [39], [51]. With this in mind, we determined KRT10 protein levels and found them basically unchanged following irradiation (Fig. 3C, D and Table S3). The additional RNA analysis confirmed this tendency toward diminished *KRT10* expression in the irradiated groups (Fig. 2, as indicated).

### The accumulation of the Ca^2+^ - binding proteins S100A8 and S100A9 correlates with the dose dependent onset of skin repair after neutron irradiation

**Figure 4:**
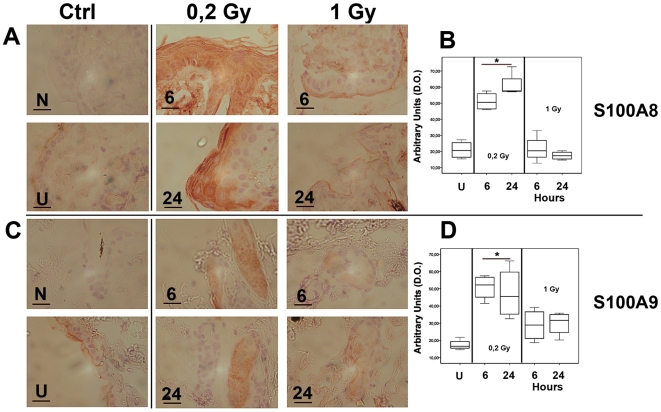
Immunoreactivity forS100A8 and S100A9. Representative images for these genes are reported in Panel A for S100A8, and Panel C for S100A9. Scale bar and abbreviations are identical to Fig. 3. Both S100A8 and S100A9 were cytoplasmic accumulated at the lower dose of 0.2 Gy group (panel A, C respectively). In the S100A8 analysis, a thickening of epidermis was evident as compared with sham and 1 Gy irradiated mice, meanwhile S100A9 was mainly detectable at the hair follicle level. Arbitrary densitometric units of protein expression are plotted as whisker box in panel B, D, with limited individual variation within the groups. Statistical analysis showed significantly (p<0,05) higher level for the group of mice treated with 0,2 Gy irradiation (*).

As shown in Fig. 4A,C for S100A8 and S100A9, both proteins were accumulated, in response to 0.2 Gy, at 6 and 24 hours after irradiation. S100A8 showed an uniform distribution in the epidermis which appears thickened, as compared to sham and 1 Gy irradiated mice. The S100A9 gene accumulation was less evident and more localized to hair follicles, as compared to epidermis (Fig. 4C), partly due to a lower antibody sensitivity. Densitometric analysis depicted as whisker box plot (Fig. 4B,D) and in Table S3, showed statistically significant differences between the groups irradiated at 0.2 Gy and the sham controls with limited individual variation within the groups. RT-qPCR analysis were performed individually on these additional four mice and the fold change normalized to the housekeeping gene *GAPDH* was represented as whisker box plot (Fig. 2, as indicated). Increased RNA expression for both *S100A8* and *S100A9* genes was detected in the 0.2 Gy irradiated mice with limited levels of individual variability.

### Concerted multicellular skin reaction to ionizing radiation damage is dose-dependent

**Figure 5:**
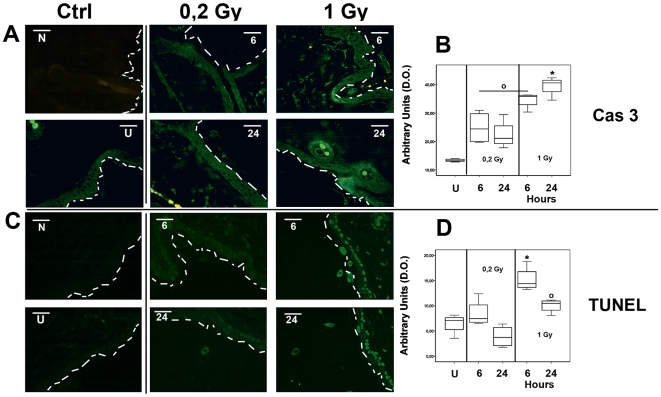
Apoptotis determination by Caspase-3 and TUNEL staining. Representative images of Cas-3 accumulation and TUNEL reaction are reported in Panel A, and C respectively. Scale bar and abbreviations are identical to Fig. 3. The negative control N for TUNEL reaction was with the omission of the enzyme mix of TUNEL reaction. The dashed white line indicates the external border of stratified epithelia. Cas-3 accumulation were cytoplasmic located and arbitrary densitometric units plotted as whisker box in panel B show statistically significant (p<0,05) higher level for the 1 Gy, 24 hours mice (*), and 1 Gy, 6 hours groups of treated mice (O) in respect of sham irradiated mice. Cas-3 accumulation shows limited individual variation within the groups. Arbitrary densitometric units of positive fluorescent TUNEL reaction are plotted as whisker box in Panel E. Statistical analysis showed a significantly (p<0,05) higher reactivity for the 1 Gy, 6 hours treated mice (*) compared to all either 0.2 Gy treated mice. The 1 Gy, 24 hours group showed a significantly (p<0,05) higher level reactivity as compared with sham irradiated and 0.2 Gy, 24 hours treated mice (O). TUNEL reaction shows limited individual variation within the groups.

**Figure 6:**
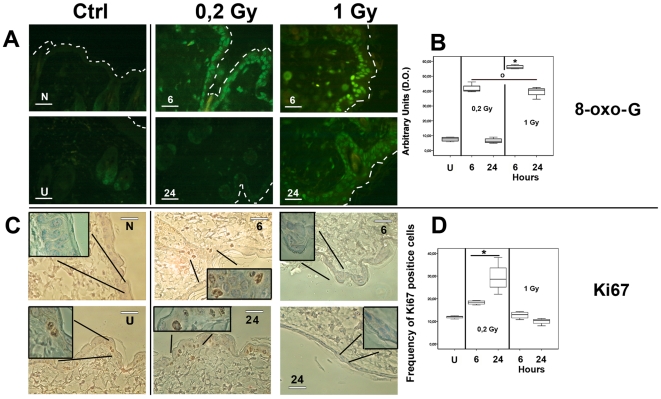
Oxydative DNA damage determination by 8-oxo-G reaction and proliferation rate by Ki67 immunohistochemistry. Representative images of 8-oxo-G reaction and Ki67 immunohistochemistry are reported in Panel A, and C respectively. Scale bar and abbreviations are identical to Fig. 3 for Ki67. Negative control N for 8-oxo-G reactivity is obtained with omission of the probe mix. Reactivity of the 8-oxo-G was nuclear in all samples. Worth noting, at 24 hours after irradiation, 1 Gy sample showed higher reactivity and a spread localization of damaged nuclei across stratified epithelia. On the contrary, 24 hours after 0.2 Gy exposure showed faintly positive nuclei that were localized along the basal layer. Arbitrary densitometric units of positive fluorescent 8-oxo-G reaction are plotted as whisker box in Panel B. Statistical analysis showed a significantly (p<0,05) higher reactivity for 1 Gy in the 6 hours mice (*) with respect to all other tested mice. The 0,2 Gy, in the 6 hour treated mice and 1 Gy, in the 24 hour treated mice were grouped to show a significantly (p<0,05) higher reactivity as compared with sham irradiated and 0.2 Gy, 24 hours group of mice (O). The 8-oxo-G reaction showed limited individual variation within the groups. Proliferation was determined by Ki67 nuclear localization in irradiated samples. Positive nuclei were scored following contrast phase microscopy visualization in oil immersion at 100x magnification, as shown as an inset in all samples. The frequency of positive Ki67 labeled cells are plotted as whisker box in Panel D. Statistical analysis showed a significantly (p<0,05) higher reactivity in the 0.2 Gy, 24 hours mice (*) with respect to all others treated mice. The 0.2 Gy, 6 hours treated mice and the 1 Gy, 6 hours were grouped to show a significantly (p<0,05) higher reactivity level with respect to sham irradiated and 1 Gy, 24 hours mice (O) (Panel C). The Ki67 immunohistochemistry reaction showed limited individual variation within the groups.

Cytoplasmic pro-apoptotic Caspase-3 expression and TUNEL reaction were higher at the 1 Gy, 24 hours following exposure (Fig. 5A,C and Table S3). The 0.2 Gy group showed a significantly lower level of apoptosis, as compared with 1 Gy irradiated mice, although the expression level of pro-apoptotic Caspase-3 and TUNEL determination showed apoptosis 6 hours following exposure (whisker box plot in Fig. 5B,D). These results were in agreement with the trend of p21 expression detected both by microarray analysis (Table S1), and by RT-qPCR performed on the additional four mice for each experimental group (Fig. 2, as indicated). In Fig. 6A,B, the detection of 8-oxo-G residues demonstrated a significantly higher level of damaged DNA after 6 hours at both irradiation doses. At 24 hours after 0.2 Gy delivery, the level of 8-oxo-G residues dropped dramatically down to the level of sham irradiated control, while it remained significantly higher at 1 Gy exposure (Table S3). These findings were indirectly supported by the expression of the *HMOX-1* gene, as reported in the Table S1 and in Fig. 2. The fold change of *HMOX-1* gene normalized to the level of the housekeeping gene *GADPH* is represented as whisker box plot. A significantly increased *HMOX-1* mRNA level was detected in the 0.2 Gy group of irradiated mice with a limited range of individual variability. Ki67 accumulation has been shown to correlate with proliferation rate in damaged skin tissues. As shown in Fig. 6C, a significantly higher number of positive cells was present at 24 hours after 0.2 Gy delivery, (frequency of positive cells as reported in Fig. 6D). The data (95% confidence interval of the max-min median value showed 22.3–36.4 for controls; 36.4–40.0 for 0.2 Gy at 6 hours; 55.3–67.8 for 0.2 Gy at 24 hours; 29.2–34.7 for 1 Gy at 6 hours; 22.8–27.3 for 1 Gy at 24 hours). According to the apoptosis determination, a low number of asynchronous cycling cells were still proliferating at 6 hours after either dose of irradiation. However, at 24 hours post exposure, only not apoptotic cells could proliferate to regenerate injured tissue. These cells were present exclusively in the 0.2 Gy experimental group.

## Discussion

Meta-analysis of microarray data demonstrates that cell lines, losing the organization required to form tissues, express both a smaller proportion of genes, and a different set of clustered genes, as compared to that observed in intact tissues in vivo [52]. Furthermore, although skin three-dimensional systems increase the fidelity of the tissues stress response [37], [53], transcriptome of such systems only partially overlap with human skin biopsy expression profiles; moreover such systems never replicate great variation among individual responses to radiation which is not predictable [22], [24], [54]. The differential modulation of these genes in the epidermis mimics the activation of the onset of re-epithelialization and a skin self-renewal processes. The timing of S100A8 protein accumulation after 0.2 Gy irradiation is consistent with previous experimental *in vivo* results on damaged human skin [55] which also showed absence of a dominant post-transcriptional control. Genes S100A8 and S100A9, frequently co-expressed and forming a heterodimer, belong to a multigenic and multifunctional family of calcium-binding proteins, that has been identified in several inflammatory skin conditions such as psoriasis, atopy, and cancer [56]–[58]. In agreement with our data, S100A8 and S100A9 were previously shown to be present at low levels in normal skin [59]. However, they were co-localized and clustered with various members of epidermal differentiation markers, and up-regulated during differentiation [58]. Thus, the dose dependent differential up-regulation of S100A8 and S100A9, synchronous with the onset of skin repair and not associated with skin differentiation, could be held accountable for the multi-functional roles played by the S100 family in skin tissue. The multi-functional role of the S100 family includes apoptosis inhibition [60] and some of its members have been found to be activated by reactive oxygen species (ROS) [61]. Since both of these phenomena are involved in biological effects of ionizing radiation, we analyzed apoptosis (immunohistological determination of Caspase-3 and TUNEL activation), oxidative DNA damage (accumulation of 8-oxo-G residues), and proliferation rates (immunohistological determination of Ki67 nuclear antigen) in the experimental samples. Only mice exposed to 0.2 Gy showed a substantial lack of apoptosis and oxidized DNA after 24 hours. Although mouse skin does not completely resembles the human skin, these findings represent a novel contribution to the molecular diagnostic scenario of the regenerative processes of skin after high LET irradiation, suggesting a multicellular program of response to radiation damage that includes surveillance for damaged cells, which is independent from DNA repair processes. Up-regulation of the oxidative stress plays an important role in this context. Homeostatic cellular functions require tight controls of the redox environment, since a high level of ROS can lead to disorders, and an imbalance between oxidant and antioxidant potential is detrimental to cellular life [62], [63]. The up-regulation of ROS scavengers, such as catalase and Cu-Zn superoxide dismutase (SOD) enzymes, inhibits the expression of stress-responding regulatory genes (e.g. p21). These can then induce genetic instability [64]. A higher level of ROS has been previously ascribed to cell-to-cell communications and damage transmission in the context of tissue cross-talk [1]. The almost complete depletion of oxidized DNA which we exclusively observe at 24 hours after 0.2 Gy, could be part of a redox skin's strategy to rescue tissue integrity, as suggested also by the indirect evidence of the differential over expression of *HMOX-1* only in the 0.2 Gy irradiated mice. HMOX-1 is an inducible isoform among the HMOX gene family members which is induced not only by its substrate haem, but also by exposure to a wide variety of stressful stimuli. Most of the known HMOX-1 inducers stimulate the production of ROS or lead to depletion of glutathione (GSH) levels, suggesting the involvement of HMOX-1 activity in cellular protection against oxidative stress. Interestingly, HMOX-1 has been as described over expressed also at the early stage of wound repair where it was related to skin proliferation [65]. Both repair and proliferation are apparently present only in the 0.2 Gy irradiated mice, and absent in 1 Gy irradiated mice in which the level of HMOX-1 RNA is diminished.

In a recent study, persistence of damaged DNA has been demonstrated in mouse skin irradiated with high doses of X rays. The authors argue that the persistence of clusters of damaged DNA should be considered as evidence of chronic oxidative diseases, rather than ascribed to residual DNA damage clusters formed by acute irradiation [66], and a late residual of damaged DNA on mouse skin has been linked to dose responsiveness and prediction of radio-sensitivity *in vivo* after X irradiation, on the basis of the H2AX phosphorylation level [36]. In high LET irradiated samples, however, one would expect persistent DNA damage sites caused primarily by the clustered DNA damage. In our research, we noted the absence of residual clusters of damaged DNA after 0.2 Gy exposure. This phenomenon could be achieved by elimination of damaged cells, which it may be a result of over expression of S100A8 and S100A9 proteins. Recently, the up-regulation of S100 gene family was associated with cellular migration in numerous systems, such as early-stage non-small lung cancer distant metastasis [67], and human keratinocyte trans-activation by the cytokine IL-22 to drive the migration of renewed cells from basal to more differentiated skin layers [68].

It is widely accepted that elimination of damaged cells is the principal protection system of the genome, and the two main mechanisms involved in this task are apoptosis and lack of activation of cell defense mechanisms, when the dose or dose rate are very low, the repair is absent, and the damaged cells are eliminated by mitotic death [6]. In the present study, apoptosis and elimination of damaged cells by mitotic death are associated exclusively with the 1 Gy dose. Since X-irradiation at a similar doses provokes residual clusters of damaged DNA in mice [36], [66], and a different cluster of genes is activated in human biopsies [69], [70], the differential cell reactions seen in mice after 0.2 Gy dose could depend on the quality of irradiation and neutron microdosimetry [9].

One possible physical basis for different effects of different radiation qualities, or relative biological effectiveness, is the differences in spatial distribution of the biological lesions produced by radiation [71]. Microdosimetry which is a conceptual framework used for the analysis of microscopic distribution of energy in irradiated matter, can be used to investigate the physical basis of primary lesions induced by radiation. When neutrons irradiate living tissues, mammalian cells experience ionization and excitation events due to charged particles set in motion by the neutrons. The total amount of energy absorbed in a given site, because of the passage of a single ionizing particle through or close to the site, depends on the target size and on the particle electrical charge and velocity. When the absorbed dose is lower (i.e. 0.2 Gy), the number of biophysical events in irradiated cells decreases. Some holds true for the proportion of cells which are traversed by ionizing particles, albeit the damage caused by one single neutron is always the same [72]. Obviously, the probability of delivering an irreparable DNA damage is higher when numerous double-strand breaks are simultaneously present in the same nucleus. Radiobiological measurements have pointed out that clusterized ionization events induce sever primary damage, which is less efficiently repaired with respect to that one that is induced by less clusterized ionization events. How big has to be the cluster size, to give significantly different radiobiological results, and large the critical site size where it occurs, is matter of scientific investigation. As calculated in Appendix S1 and shown in Fig. S2, the cumulative probability ***F_2_*** to have two simultaneous or more ionization events in a 1–2 µm sized biological (chromosomal level) site is drastically lower for the 0.2 Gy as compared to 1 Gy dose level, and that happens also for radiobiological data, which are qualitatively different at 1 Gy with respect to 0.2 Gy of absorbed dose, in spite the fact that the radiation field is always the same. It is remarkable observing that such a coincidence wouldn't occur if the critical site size were smaller than 1 µm or bigger than 2 µm. This prediction provides insight into the interpretation of the differential tissue's reaction at the two doses. In our *in vivo* models, the ability to trigger whole tissue defense could strongly depend upon the proportion of cells that received multiple clustered DNA damage. Our data also shows *in vivo* evidence of a regenerative skin processes executed through cell turnover and independently from DNA repair-associated pathways. These findings do not completely agree with experiments performed on phantom and survival curves of V79 cell line previously used as biological dosimeter to calculate the relative biological effectiveness (RBE) of 14 MeV neutrons. These data showed a higher RBE for the lower dose as compared with the higher dose [42]. However, what occur *in vitro* with a cell line may only approximate the whole tissue's response to radiation damage. In the future, this study could be extended to identification of the skin's regenerative processes after irradiation at later times and after a wider range of doses of low-LET and high-LET radiation including heavy-ions and protons. This type of *in vivo* studies could open a discussion and offer new insight for radiotherapists and radiation protection investigators. With regard to molecular mechanisms, it would be interesting to verify the roles of S100A8 and S100A9 genes in the skin regenerating processes after delivering radiation to specific genetically modified mouse models where those genes have been altered.

## Supporting Information

Figure S1 **Microarrays and RT-qPCR expression analysis correlation.** Comparison of microarrays and RT-qPCR expression values for selected genes. The results demonstrate good correlation (R = 0.88; p-value = 9.7E-14) between the microarrays and RT-qPCR data. GAPDH mRNA level was used as internal control in RT-qPCR analysis.
(TIFF)

Figure S2 **Nuclear microdosimetry.** Probability *F_2_* that 2 o more ionization events occur in a site of a give diameter for different absorbed doses *D*. Dashed lines: calculations from Caswell and Coyne model (see text, Ref. 46] •: calculations from measured data (see text, Ref. 45).
(TIF)

Table S1 **List of modulated genes.** List of 440 genes modulated at least 1.5 fold in two different conditions in comparison to the sham-irradiated control and filtered on the base of consistency in replicated experiments by Student t-tests (P<0.05).
(PDF)

Table S2 **Functional Annotation Clustering of modulated genes.** Functional Annotation Clustering report of 440 genes modulated at least 1.5 fold in two different conditions in comparison to the sham-irradiated control using DAVID (see text, Ref. 49) and (http://david.abcc.ncifcrf.gov/home.jsp) web tool. The grouping algorithm is based on the hypothesis that similar annotations should have similar gene members.
(PDF)

Table S3 **Immunohistochemistry imaging analysis.** Immunoreactivities expresssed as arbitrary densitometric units 95% confidential interval min-max median value. After normalization for the intensity of background staining, at least four areas for each slide were scored by ImageJ dedicated software for covering of the epidermal layer and hair follicles.
(PDF)

Appendix S1 **Algorithms for neutron microdosimetry.** Multiple ionization event numbers (***n*** *)* for different site sizes and the absorbed dose (***D*** *)* values calculated by using microdosimetric algorithms (see Fig. S1 and Ref. 45, 46).
(DOC)

Appendix S2 ** Radiobiological units and abbreviations used in the text.**
(DOC)
