# Supplementary figures and images for: Dose-Dependent Onset of Regenerative Program in Neutron Irradiated Mouse Skin
Source: PLoS One. 2011 Apr 27;6(4):e19242. doi: 10.1371/journal.pone.0019242 (PMC3083422; doi:10.1371/journal.pone.0019242)

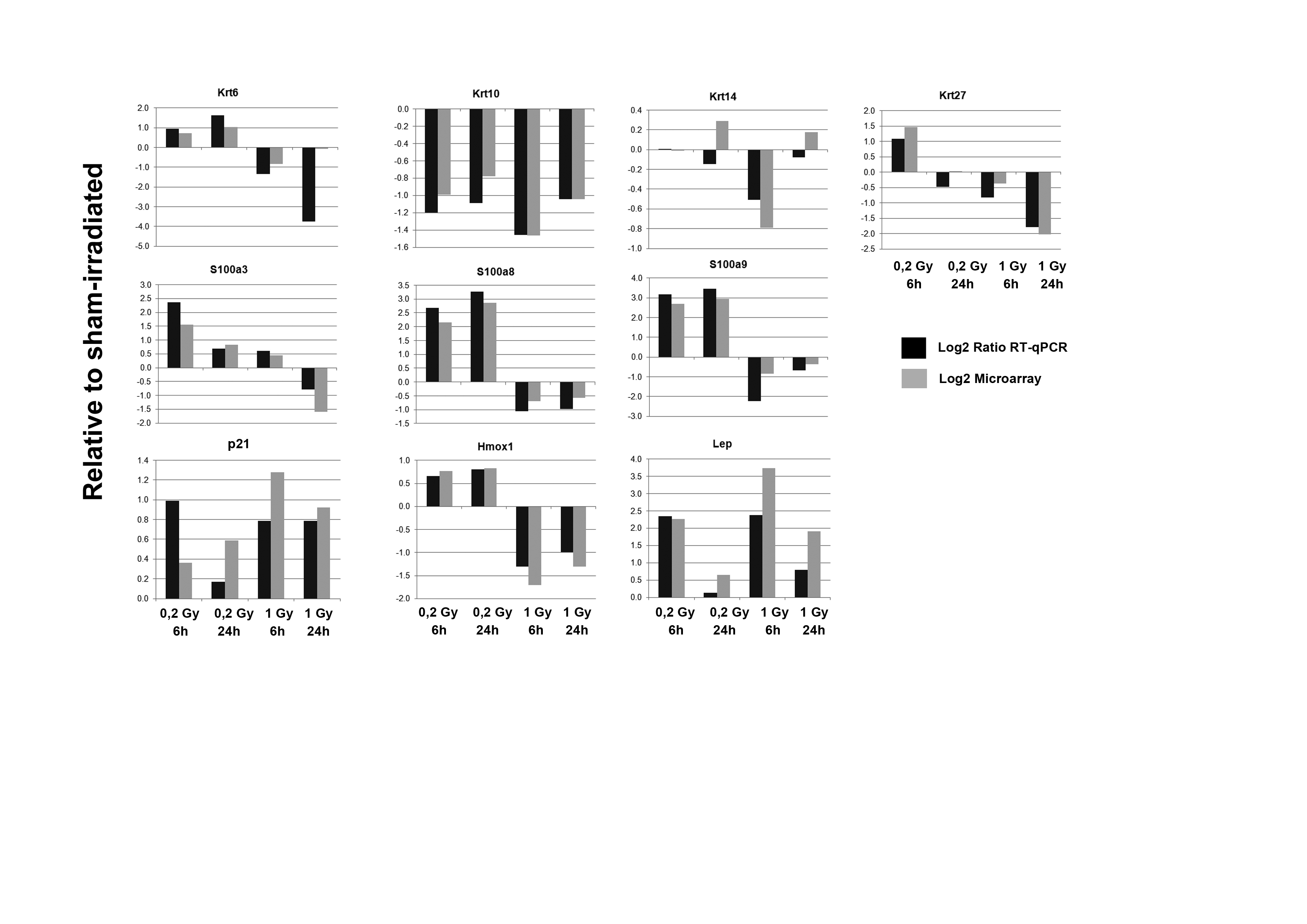

Supplement: Figure S1 — Microarrays and RT-qPCR expression analysis correlation. Comparison of microarrays and RT-qPCR expression values for selected genes. The results demonstrate good correlation (R = 0.88; p-value = 9.7E-14) between the microarrays and RT-qPCR data. GAPDH mRNA level was used as internal control in RT-qPCR analysis. (TIFF) [file pone.0019242.s001.tiff]
