# Supplementary material for: Dose-Dependent Onset of Regenerative Program in Neutron Irradiated Mouse Skin
Source: PLoS One. 2011 Apr 27;6(4):e19242. doi: 10.1371/journal.pone.0019242 (PMC3083422; doi:10.1371/journal.pone.0019242)
Supplement: Table S1 — List of modulated genes. List of 440 genes modulated at least 1.5 fold in two different conditions in comparison to the sham-irradiated control and filtered on the base of consistency in replicated experiments by Student t-tests (P<0.05). (PDF) [file pone.0019242.s003.pdf]

TABLE S1. List of 440 genes modulated.

RefSeq	Gene Symbol	Name	Log2 Ratio			
			0.2 Gy 6h	0.2 Gy 24h	1 Gy 6h	1 Gy 24h
XM_001474332	**1110025L11Rik**	RIKEN cDNA 1110025L11 gene	1,42	0,46	0,18	-1,74
NM_172401	**1110057K04Rik**	RIKEN cDNA 1110057K04 gene	-0,64	-0,84	-0,59	-0,14
NM_025427	**1190002H23Rik**	RIKEN cDNA 1190002H23 gene	0,45	0,65	1,11	0,87
XR_005155	**A030005K14Rik**	RIKEN cDNA A030005K14 gene	1,74	1,10	0,09	-1,69
NM_153416	**Aaas**	achalasia, adrenocortical insufficiency, alacrimia	NA	0,48	1,26	0,90
NM_207668	**Acpp**	acid phosphatase, prostate	0,63	-0,31	NA	-1,12
NM_080575	**Acss1**	acyl-CoA synthetase short-chain family member 1	-0,77	-0,16	0,65	0,48
NM_009608	**Actc1**	actin, alpha, cardiac muscle 1	-1,67	-0,56	-1,93	-2,01
NM_019673	**Actl6a**	actin-like 6A	0,46	-0,25	-0,84	-0,79
NM_009627	**Adm**	adrenomedullin	0,83	0,86	NA	NA
NM_001008533	**Adora1**	adenosine A1 receptor	-0,98	0,21	-1,95	0,50
NM_026531	**Aen**	apoptosis enhancing nuclease	0,57	0,56	1,45	1,21
XM_980696	**AI607873**	expressed sequence AI607873	0,52	-0,14	-1,10	-1,07
NM_019467	**Aif1**	allograft inflammatory factor 1	-0,97	-0,46	-0,74	-0,01
NM_013467	**Aldh1a1**	aldehyde dehydrogenase family 1, subfamily A1	-0,05	0,16	1,55	1,29
NM_145684	**Alox12e**	arachidonate lipoxygenase, epidermal	-0,84	-0,97	-0,59	-0,41
NM_013468	**Ankrd1**	ankyrin repeat domain 1 (cardiac muscle)	0,09	1,14	-1,56	-0,03
NM_020033	**Ankrd2**	ankyrin repeat domain 2 (stretch responsive muscle)	NA	1,77	NA	1,59
NM_130889	**Anp32b**	acidic (leucine-rich) nuclear phosphoprotein 32 family, member B	0,65	0,20	-0,38	-0,64
NM_009673	**Anxa5**	annexin A5	-0,24	-0,38	-0,85	-0,63
NM_016689	**Aqp3**	aquaporin 3	0,69	0,20	NA	-0,76
NM_007476	**Arf1**	ADP-ribosylation factor 1	0,07	-0,42	-1,33	-1,17
NM_025711	**Aspn**	asporin	-0,81	-0,95	-0,83	-0,68
NM_178405	**Atp1a2**	ATPase, Na+/K+ transporting, alpha 2 polypeptide	-0,92	0,12	0,63	NA
NM_007502	**Atp1b3**	ATPase, Na+/K+ transporting, beta 3 polypeptide	0,27	-0,64	-1,19	-1,32
NM_177746	**Awat2**	acyl-CoA wax alcohol acyltransferase 2	-0,86	-0,74	0,01	0,20
NM_133359	**AY026312**	cDNA sequence AY026312	1,99	0,50	0,32	-1,68
NM_130452	**Bbox1**	butyrobetaine (gamma), 2-oxoglutarate dioxygenase 1 (gamma-butyrobetaine hydroxylase)	-1,04	-0,71	-0,19	-0,12
NM_145635	**BC054059**	cDNA sequence BC054059	NA	0,05	0,75	0,60
NM_016916	**Blcap**	bladder cancer associated protein homolog (human)	-0,23	0,37	0,62	0,61
NM_144938	**C1s**	complement component 1, s subcomponent	-0,37	-0,81	NA	-1,30
NM_025451	**Camk2n1**	calcium/calmodulin-dependent protein kinase II inhibitor 1	-0,55	0,10	0,90	0,77
NM_009811	**Casp6**	caspase 6	0,01	-0,60	-0,65	-0,57
NM_009813	**Casq1**	calsequestrin 1	-1,02	-0,50	NA	-1,08
NM_013803	**Casr**	calcium-sensing receptor	NA	0,19	0,61	0,68
NM_011333	**Ccl2**	chemokine (C-C motif) ligand 2	1,25	1,55	-0,44	0,23

NM_013653	**Ccl5**	chemokine (C-C motif) ligand 5	-0,64	-1,06	-0,59	-0,84
NM_011338	**Ccl9**	chemokine (C-C motif) ligand 9	0,92	0,90	0,73	-0,12
NM_009831	**Ccng1**	cyclin G1	-0,27	0,40	1,28	0,78
NM_009915	**Ccr2**	chemokine (C-C motif) receptor 2	NA	-0,60	-1,33	-0,79
NM_009840	**Cct8**	chaperonin containing Tcp1, subunit 8 (theta)	0,34	0,02	-0,70	-0,66
NM_009841	**Cd14**	CD14 antigen	1,54	0,72	NA	-0,89
NM_130904	**Cd209d**	CD209d antigen	-0,26	0,20	0,83	1,02
NM_007651	**Cd53**	CD53 antigen	NA	-0,35	-1,03	-1,11
NM_001042605	**Cd74**	CD74 antigen (invariant polypeptide of major histocompatibility complex, class II antigen-associated)	-1,01	-0,81	-0,16	-0,52
NM_010545	**Cd74**	CD74 antigen (invariant polypeptide of major histocompatibility complex, class II antigen-associated)	-1,09	-0,88	-0,22	-0,27
NM_007659	**Cdk1**	cyclin-dependent kinase 1	0,93	0,14	-0,36	-0,67
NM_007669	**Cdkn1a**	cyclin-dependent kinase inhibitor 1A (P21)	0,36	0,59	1,28	0,92
NM_133869	**Cept1**	choline/ethanolaminephosphotransferase 1	0,21	-0,16	-0,86	-0,66
NM_013459	**Cfd**	complement factor D (adipsin)	0,18	0,48	1,63	0,80
NM_026929	**Chac1**	ChaC, cation transport regulator-like 1 (E. coli)	0,64	-0,05	0,13	-1,20
NM_023850	**Chst1**	carbohydrate (keratan sulfate Gal-6) sulfotransferase 1	NA	0,31	1,98	1,44
NM_178373	**Cidec**	cell death-inducing DFFA-like effector c	0,95	0,53	2,02	NA
NM_173385	**Cilp**	cartilage intermediate layer protein, nucleotide pyrophosphohydrolase	-0,54	-0,27	-0,87	-0,62
NM_175451	**Ckap4**	cytoskeleton-associated protein 4	0,53	0,37	-2,09	-1,62
NM_198415	**Ckmt2**	creatine kinase, mitochondrial 2	NA	0,72	0,79	0,62
NM_009899	**Clca1**	chloride channel calcium activated 1	-1,52	-0,98	-0,83	-0,43
NM_011999	**Clec4a2**	C-type lectin domain family 4, member a2	NA	-0,81	-0,78	-0,81
NM_153197	**Clec4a3**	C-type lectin domain family 4, member a3	-0,91	-0,66	-1,18	-0,77
NM_027218	**Clec4b1**	C-type lectin domain family 4, member b1	NA	-1,05	-1,01	-1,02
NM_010819	**Clec4d**	C-type lectin domain family 4, member d	1,20	0,95	0,33	-0,38
NM_020001	**Clec4n**	C-type lectin domain family 4, member n	-0,59	-0,74	-0,87	-0,71
NM_007742	**Col1a1**	collagen, type I, alpha 1	-0,57	-0,30	-0,82	-0,74
NM_015734	**Col5a1**	collagen, type V, alpha 1	-0,65	-0,53	-0,61	-0,68
NM_146007	**Col6a2**	collagen, type VI, alpha 2	-0,68	-0,47	-0,44	-0,71
NM_147778	**Commd3**	COMM domain containing 3	0,29	0,22	0,68	0,82
NM_015827	**Copb2**	coatomer protein complex, subunit beta 2 (beta prime)	-0,11	-0,19	-0,65	-0,65
NM_013496	**Crabp1**	cellular retinoic acid binding protein I	NA	0,02	-0,82	0,60
NM_019922	**Crtap**	cartilage associated protein	-0,28	-0,34	-0,75	-0,61
NM_009964	**Cryab**	crystallin, alpha B	-0,10	0,91	0,34	1,08
NM_021351	**Cryba4**	crystallin, beta A4	1,20	0,52	0,84	NA
NM_016669	**Crym**	crystallin, mu	1,35	0,13	0,13	-1,85
NM_009976	**Cst3**	cystatin C	-0,72	-0,71	-0,09	-0,02
NM_026778	**Cthrc1**	collagen triple helix repeat containing 1	-0,46	-0,09	-0,71	-1,15
NM_009982	**Ctsc**	cathepsin C	0,11	-0,38	-0,85	-0,93
NM_007802	**Ctsk**	cathepsin K	-0,50	-0,63	-0,84	-0,46

NM_021281	**Ctss**	cathepsin S	-0,43	-0,58	-0,75	-0,92
NM_023158	**Cxcl16**	chemokine (C-X-C motif) ligand 16	-0,60	-0,91	-0,69	-0,66
NM_009140	**Cxcl2**	chemokine (C-X-C motif) ligand 2	1,94	1,68	NA	-0,98
NM_007814	**Cyp2b19**	cytochrome P450, family 2, subfamily b, polypeptide 19	-0,80	-0,50	-0,92	-0,20
NM_021282	**Cyp2e1**	cytochrome P450, family 2, subfamily e, polypeptide 1	0,13	1,09	1,55	0,48
NM_029653	**Dapk1**	death associated protein kinase 1	NA	-0,06	1,10	1,02
NM_026428	**Dcxr**	dicarbonyl L-xylulose reductase	NA	NA	1,61	0,83
NM_007840	**Ddx5**	DEAD (Asp-Glu-Ala-Asp) box polypeptide 5	0,37	-0,27	-0,68	-0,81
NM_183026	**Defb14**	defensin beta 14	NA	1,41	NA	0,86
NM_007879	**Drg1**	developmentally regulated GTP binding protein 1	1,79	1,67	0,90	NA
NM_019819	**Dusp14**	dual specificity phosphatase 14	0,80	-0,22	-0,09	-1,30
NM_019398	**Ear5**	eosinophil-associated, ribonuclease A family, member 5	-0,67	-0,35	-0,66	0,37
NM_001012324	**Ecm2**	extracellular matrix protein 2, female organ and adipocyte specific	-0,99	-0,86	-0,64	-0,51
NM_028133	**Egln3**	EGL nine homolog 3 (C. elegans)	-1,04	-0,57	-0,71	-0,67
NM_010118	**Egr2**	early growth response 2	-0,37	-0,64	-0,49	-0,73
NM_007915	**Ei24**	etoposide induced 2.4 mRNA	-0,02	0,17	0,94	0,69
NM_145380	**Eif3m**	eukaryotic translation initiation factor 3, subunit M	0,32	-0,47	-0,69	-0,79
NM_130450	**Elovl6**	ELOVL family member 6, elongation of long chain fatty acids (yeast)	-0,81	-0,65	-1,71	-0,76
NM_010128	**Emp1**	epithelial membrane protein 1	0,71	-0,09	-0,69	-0,83
NM_023580	**Epha1**	Eph receptor A1	0,32	0,13	-0,77	-0,94
NM_010145	**Ephx1**	epoxide hydrolase 1, microsomal	-0,05	0,32	1,28	1,17
NM_010161	**Evi2a**	ecotropic viral integration site 2a	-0,78	-0,51	-0,64	-0,45
NM_010174	**Fabp3**	fatty acid binding protein 3, muscle and heart	-0,36	0,61	0,98	0,91
NM_024406	**Fabp4**	fatty acid binding protein 4, adipocyte	0,90	0,68	1,71	0,92
NM_026635	**Fam96a**	family with sequence similarity 96, member A	-0,04	-0,18	-0,61	-0,62
NM_007993	**Fbn1**	fibrillin 1	-0,21	-0,25	-0,60	-0,81
NM_019395	**Fbp1**	fructose bisphosphatase 1	2,50	1,37	NA	NA
NM_015791	**Fbxo8**	F-box protein 8	-0,21	-0,66	-0,50	-0,60
NM_001077189	**Fcgr2b**	Fc receptor, IgG, low affinity IIb	-0,15	-0,41	-0,73	-0,65
NM_008013	**Fgl2**	fibrinogen-like protein 2	0,74	-0,15	-1,24	-1,34
NM_153573	**Fkbp14**	FK506 binding protein 14	-0,74	-0,43	-0,73	-0,47
XM_001481265	**Flg**	filaggrin	-0,83	-0,54	-2,05	-1,95
NM_010228	**Flt1**	FMS-like tyrosine kinase 1	0,31	0,47	0,74	0,79
NM_010233	**Fn1**	Fibronectin 1	-0,46	-0,63	-0,94	-1,40
NM_010234	**Fos**	FBJ osteosarcoma oncogene	0,17	-0,69	0,32	-0,73
NM_199068	**Foxk1**	forkhead box K1	NA	0,05	-0,88	-1,14
NM_008059	**G0s2**	G0/G1 switch gene 2	0,80	0,78	2,60	1,04
NM_007836	**Gadd45a**	growth arrest and DNA-damage-inducible 45 alpha	0,32	0,55	-1,01	-0,95
NM_010288	**Gja1**	gap junction protein, alpha 1	0,87	-0,10	-0,74	-0,99
NM_008125	**Gjb2**	gap junction protein, beta 2	1,24	0,27	-0,53	-1,57

NM_008128	**Gjb6**	gap junction protein, beta 6	1,09	0,02	-0,78	-1,79
NM_153803	**Glb1l2**	galactosidase, beta 1-like 2	0,13	-0,18	1,29	0,64
XR_031623	**Gm10075**	predicted gene 10075	0,19	-0,29	-1,13	-1,02
XM_001481215	**Gm10184**	predicted gene 10184	0,99	-0,02	-1,02	-0,91
XM_001474297	**Gm10228**	predicted gene 10228	1,31	0,31	0,38	-1,94
XM_001474256	**Gm10229**	predicted gene 10229	1,57	0,44	0,54	-2,04
XM_001476503	**Gm10291**	predicted gene 10291	-0,45	-0,42	-0,82	-0,88
XM_001473172	**Gm10293**	predicted gene 10293	-0,66	-0,61	-0,84	-1,08
XR_033442	**Gm10540**	predicted gene 10540	-0,08	-0,22	-0,78	-0,62
XM_001478013	**Gm10698**	predicted gene 10698	0,03	-0,47	-1,32	-0,89
XR_032746	**Gm11453**	predicted gene 11453	NA	-0,35	-0,65	-0,74
XM_001472187	**Gm11554**	predicted gene 11554	0,99	0,26	-0,21	-1,63
XM_001477815	**Gm11555**	predicted gene 11555	0,75	0,22	0,12	-0,88
XR_030645	**Gm11565**	predicted gene 11565	2,06	1,06	NA	NA
XM_001472217	**Gm11568**	predicted gene 11568	1,02	-0,35	-0,63	-2,31
XM_001474519	**Gm11568**	predicted gene 11568	0,83	-0,09	-0,07	-1,85
XR_031894	**Gm11658**	predicted gene 11658	-0,25	-0,16	-0,60	-0,67
XM_001477731	**Gm11938**	predicted gene 11938	0,96	0,28	0,30	-0,99
XR_032345	**Gm11960**	predicted gene 11960	-0,31	-0,35	-0,88	-0,67
XR_032243	**Gm12009**	predicted gene 12009	0,15	-0,03	-0,75	-0,78
XR_031980	**Gm12183**	predicted gene 12183	0,67	-0,87	NA	NA
XM_989911	**Gm12231**	predicted gene 12231	NA	-0,31	-0,63	-0,60
XM_890719	**Gm12271**	predicted gene 12271	0,41	-0,07	-0,81	-0,66
XR_033516	**Gm12416**	predicted gene 12416	-0,55	-0,60	-1,05	-1,05
XR_033065	**Gm12587**	predicted gene 12587	0,37	-0,20	-1,17	-1,04
XR_031401	**Gm12715**	predicted gene 12715	0,68	0,00	-0,87	-0,57
XR_032022	**Gm12813**	predicted gene 12813	NA	0,08	0,73	0,74
XR_032273	**Gm12960**	predicted gene 12960	0,31	-0,01	-0,98	-0,75
NM_001081248	**Gm13177**	predicted gene 13177	-0,32	-0,61	-0,85	-0,29
XR_030808	**Gm13331**	predicted gene 13331	0,36	-0,25	-0,69	-0,65
XM_001472735	**Gm13464**	predicted gene 13464	-0,32	-0,69	-0,85	-1,05
XR_033935	**Gm13529**	predicted gene 13529	0,10	-0,07	-0,63	-0,73
XR_034973	**Gm14111**	predicted gene 14111	-0,58	-0,33	-0,71	-0,80
XR_031372	**Gm14730**	predicted gene 14730	-0,06	-0,06	-0,88	-0,64
XR_035647	**Gm15294**	predicted gene 15294	-0,44	-0,19	-0,71	-0,85
XM_001472867	**Gm15452**	predicted gene 15452	0,71	-0,19	NA	-1,24
XR_030737	**Gm15470**	predicted gene 15470	0,64	0,73	-0,28	-0,27
XM_001472395	**Gm16374**	predicted gene 16374	-0,49	-0,37	-0,91	-0,87
XR_030800	**Gm2308**	predicted gene 2308	-0,42	-0,42	-0,71	-0,75
XM_001473743	**Gm2451**	predicted gene 2451	-0,47	-0,55	-1,03	-0,79

XR_031402	**Gm2509**	predicted gene 2509	0,32	-0,25	-1,13	-1,14
XR_031094	**Gm2546**	predicted gene 2546	-0,41	-0,44	-1,00	-0,89
XM_001474369	**Gm2606**	predicted gene 2606	-0,47	-0,30	-0,71	-0,78
XR_031562	**Gm2607**	predicted gene 2607	0,29	-0,24	-1,00	-0,84
XM_001475435	**Gm2802**	predicted gene 2802	0,59	-0,12	-0,81	-0,95
XR_031404	**Gm3148**	predicted gene 3148	0,26	-0,07	-0,90	-0,73
XM_001475962	**Gm3200**	predicted gene 3200	-0,61	-0,33	-0,77	-0,83
XR_031443	**Gm3226**	predicted gene 3226	0,41	-0,20	-0,84	-0,73
XM_001476937	**Gm3380**	predicted gene 3380	0,69	0,15	-0,15	-0,68
XR_031059	**Gm3695**	predicted gene 3695	-0,37	-0,25	-0,63	-0,61
XM_001476909	**Gm4130**	predicted gene 4130	0,57	-0,62	-1,06	-1,26
XM_001480790	**Gm4559**	predicted gene 4559	1,10	0,28	-0,14	-1,06
XM_001479173	**Gm4596**	predicted gene 4596	0,28	-0,48	-0,92	-0,82
NM_001034864	**Gm4907**	predicted gene 4907	-0,54	-0,35	-0,75	-0,81
XR_033797	**Gm4973**	predicted gene 4973	-0,23	-0,31	-0,67	-0,79
XR_035076	**Gm5069**	predicted gene 5069	-0,41	-0,14	-0,73	-0,60
XR_031075	**Gm5210**	predicted gene 5210	-0,36	-0,40	-0,71	-0,75
XM_895768	**Gm5469**	predicted gene 5469	-0,03	-0,17	-1,39	-1,39
XR_034364	**Gm5822**	predicted gene 5822	0,29	-0,39	-0,67	-0,59
XR_031745	**Gm5842**	predicted gene 5842	0,21	-0,11	-1,00	-0,78
XM_917495	**Gm5858**	predicted gene 5858	-0,06	-0,21	-0,85	-0,68
XR_031351	**Gm6032**	predicted gene 6032	0,39	0,91	1,09	1,15
XM_884320	**Gm6115**	predicted gene 6115	0,31	-0,18	-0,99	-0,61
XM_884506	**Gm6128**	predicted gene 6128	0,14	-0,22	-0,68	-0,65
XM_884529	**Gm6132**	predicted gene 6132	0,16	-0,33	-0,69	-0,62
XR_034780	**Gm6375**	predicted gene 6375	0,55	-0,43	-0,93	-0,97
XM_001474670	**Gm6421**	predicted gene 6421	0,13	-0,28	-0,71	-0,59
XR_032303	**Gm6542**	predicted gene 6542	0,12	-0,24	-0,72	-0,70
XR_034709	**Gm6589**	predicted gene 6589	0,28	-0,13	-0,91	-0,71
XM_894313	**Gm6747**	predicted gene 6747	0,24	-0,16	-1,04	-0,67
XR_033587	**Gm6789**	predicted gene 6789	0,40	-0,16	-0,93	-0,90
XR_032957	**Gm6947**	predicted gene 6947	0,19	-0,17	-0,76	-0,71
XR_031903	**Gm7129**	predicted gene 7129	-0,63	-0,26	-0,64	-0,63
XM_001480150	**Gm7293**	predicted gene 7293	-0,60	-0,47	-0,65	-0,75
XM_917767	**Gm7308**	predicted gene 7308	-0,32	-0,62	-0,79	-0,79
XR_030624	**Gm7422**	predicted gene 7422	0,38	-0,03	-0,91	-0,71
XR_032010	**Gm7870**	predicted gene 7870	0,09	-0,32	-0,86	-0,85
XR_032366	**Gm8554**	predicted gene 8554	0,31	-0,22	-0,82	-0,64
XR_032143	**Gm8556**	predicted gene 8556	0,27	-0,16	-0,79	-0,66
XR_033768	**Gm8594**	predicted gene 8594	0,43	0,01	-0,71	-0,75

XR_032443	**Gm8709**	predicted gene 8709	-0,53	-0,31	-0,82	-0,73
XR_030992	**Gm8756**	predicted gene 8756	-0,42	-0,45	-1,05	-1,02
XR_002080	**Gm8824**	predicted gene 8824	-0,51	-0,48	-0,79	-0,71
XR_032793	**Gm8927**	predicted gene 8927	-0,28	-0,32	-0,65	-0,70
XR_034310	**Gm9061**	predicted gene 9061	-0,35	-0,41	-0,94	-1,01
XR_031670	**Gm9115**	predicted gene 9115	0,89	0,88	-0,28	-0,03
XR_033813	**Gm9118**	predicted gene 9118	0,32	-0,37	-0,87	-1,06
XR_032065	**Gm9432**	predicted gene 9432	0,87	0,17	NA	-0,72
XM_001474241	**Gm9829**	predicted gene 9829	1,56	0,48	0,47	-1,17
XM_001478515	**Gm9844**	predicted gene 9844	-0,35	-0,32	-0,64	-0,61
NM_010271	**Gpd1**	glycerol-3-phosphate dehydrogenase 1 (soluble)	NA	0,96	0,51	0,64
NM_011823	**Gpr34**	G protein-coupled receptor 34	-1,35	-1,19	-0,90	-1,36
NM_008161	**Gpx3**	glutathione peroxidase 3	-0,67	0,11	0,79	0,35
NM_008175	**Grn**	granulin	-0,11	-0,27	-0,65	-0,70
NM_010378	**H2-Aa**	histocompatibility 2, class II antigen A, alpha	-1,12	-1,09	-0,28	-0,49
NM_207105	**H2-Ab1**	histocompatibility 2, class II antigen A, beta 1	-0,91	-1,31	-0,64	-0,84
NM_010382	**H2-Eb1**	histocompatibility 2, class II antigen E beta	-0,90	-0,81	-0,15	-0,24
NM_001001892	**H2-K1**	histocompatibility 2, K1, K region	-0,37	-0,78	-0,51	-0,61
NM_010391	**H2-Q10**	histocompatibility 2, Q region locus 10	-0,64	-0,79	-0,65	-0,45
NM_010380	**H2-Q2**	histocompatibility 2, Q region locus 2	-0,55	-0,78	-0,67	-0,64
NM_008210	**H3f3a**	H3 histone, family 3A	0,06	-0,32	-0,62	-0,64
XM_889893	**H3f3c**	H3 histone, family 3C	NA	-0,27	-0,64	-0,67
NM_008218	**Hba-a1**	hemoglobin alpha, adult chain 1	0,30	0,03	1,17	-0,74
NM_008220	**Hbb-b1**	hemoglobin, beta adult major chain	0,65	0,06	0,62	-1,56
NM_008230	**Hdc**	histidine decarboxylase	2,13	1,27	NA	NA
NM_013546	**Hebp1**	heme binding protein 1	-0,02	-0,14	0,86	0,73
XM_974905	**Hectd1**	HECT domain containing 1	0,01	-0,24	-0,99	-0,86
XM_001473104	**Hmgn4**	high mobility group nucleosomal binding domain 4	0,16	-0,12	-0,80	-0,65
NM_010442	**Hmox1**	heme oxygenase (decycling) 1	0,77	0,83	-1,70	-1,30
NM_010447	**Hnrnpa1**	heterogeneous nuclear ribonucleoprotein A1	0,56	-0,38	-0,99	-1,01
XM_001000796	**Hrnr**	hornerin	NA	-0,59	-1,90	-1,92
NM_019657	**Hsd17b12**	hydroxysteroid (17-beta) dehydrogenase 12	0,17	-0,42	-1,28	-0,95
NM_008321	**Id3**	inhibitor of DNA binding 3	0,23	0,79	0,69	NA
NM_008327	**Ifi202b**	interferon activated gene 202B	0,95	1,59	NA	0,25
NM_029803	**Ifi27l2a**	interferon, alpha-inducible protein 27 like 2A	0,34	0,97	0,32	0,72
NM_026820	**Ifitm1**	interferon induced transmembrane protein 1	0,95	0,88	0,36	0,21
NM_008342	**Igfbp2**	insulin-like growth factor binding protein 2	-0,74	NA	NA	-0,62
NM_152839	**Igj**	immunoglobulin joining chain	NA	-3,19	0,56	0,69
NM_008361	**Il1b**	interleukin 1 beta	1,99	0,94	NA	-0,42
NM_133775	**Il33**	interleukin 33	0,72	0,74	NA	NA

NM_001008700	**Il4ra**	interleukin 4 receptor, alpha	0,96	1,02	NA	0,03
NM_008439	**Khk**	ketohexokinase	NA	-0,08	0,63	0,73
NM_027910	**Klhdc3**	kelch domain containing 3	-0,22	-0,58	-0,99	-0,82
NM_010660	**Krt10**	keratin 10	-0,99	-0,78	-1,47	-1,04
NM_008469	**Krt15**	keratin 15	-0,74	-0,79	-0,31	0,05
NM_008470	**Krt16**	keratin 16	0,89	1,35	-1,33	-0,03
NM_010664	**Krt18**	keratin 18	0,43	0,99	1,49	1,38
NM_133730	**Krt25**	keratin 25	1,65	0,47	0,26	-2,03
NM_010666	**Krt27**	keratin 27	1,46	0,03	-0,37	-2,02
NM_010659	**Krt31**	keratin 31	1,74	0,48	0,64	-1,53
NM_027983	**Krt33a**	keratin 33A	1,75	0,23	0,23	-2,07
XM_904709	**Krt33b**	keratin 33B	1,60	0,65	0,15	-1,57
NM_027563	**Krt34**	keratin 34	1,42	0,23	0,40	-1,44
NM_016880	**Krt35**	keratin 35	1,75	1,06	0,53	NA
NM_008476	**Krt6a**	keratin 6A	0,71	1,04	-0,83	-0,06
NM_010669	**Krt6b**	keratin 6B	0,61	1,13	-2,28	0,22
NM_019956	**Krt71**	keratin 71	1,27	0,48	0,05	-1,29
NM_053249	**Krt82**	keratin 82	2,11	1,33	NA	NA
NM_008474	**Krt84**	keratin 84	-0,10	-0,11	-0,68	-0,82
NM_010667	**Krt86**	keratin 86	1,03	0,32	0,35	-1,07
NM_010670	**Krtap12-1**	keratin associated protein 12-1	1,00	0,75	NA	NA
NM_183189	**Krtap13-1**	keratin associated protein 13-1	1,57	0,49	0,23	-1,99
NM_013707	**Krtap14**	keratin associated protein 14	1,34	0,03	0,07	-1,81
NM_013713	**Krtap15**	keratin associated protein 15	1,21	0,08	0,15	-1,79
NM_130870	**Krtap16-1**	keratin associated protein 16-1	1,79	0,60	0,40	-1,61
NM_130873	**Krtap16-4**	keratin associated protein 16-4	2,23	0,80	0,59	-1,55
NM_130857	**Krtap16-5**	keratin associated protein 16-5	2,34	NA	0,51	-1,77
NM_130856	**Krtap16-8**	keratin associated protein 16-8	1,48	0,39	0,29	-1,77
XM_001472331	**Krtap2-4**	keratin associated protein 2-4	1,18	0,08	0,01	-1,58
XM_977282	**Krtap24-1**	keratin associated protein 24-1	2,29	1,15	NA	NA
XM_919110	**Krtap28-13**	keratin associated protein 28-13	1,98	0,94	-0,06	-1,85
NM_025720	**Krtap3-2**	keratin associated protein 3-2	1,08	0,09	-0,01	-1,49
NM_025524	**Krtap3-3**	keratin associated protein 3-3	1,48	0,45	0,03	-1,66
NM_001013823	**Krtap4-16**	keratin associated protein 4-16	1,06	0,10	0,13	-1,65
NM_026807	**Krtap4-2**	keratin associated protein 4-2	1,32	0,25	-0,22	-1,55
NM_029613	**Krtap4-7**	keratin associated protein 4-7	1,38	0,25	0,06	-1,98
NM_027844	**Krtap5-2**	keratin associated protein 5-2	1,51	0,52	0,08	-1,17
NM_015809	**Krtap5-4**	keratin associated protein 5-4	1,58	0,98	0,10	-0,89
NM_010672	**Krtap6-1**	keratin associated protein 6-1	1,45	0,58	0,29	-1,55
NM_010673	**Krtap6-2**	keratin associated protein 6-2	1,38	0,74	0,47	-1,32

XM_001474351	**Krtap6-3**	keratin associated protein 6-3	0,44	1,53	0,81	-0,57
XM_001474473	**Krtap7-1**	keratin associated protein 7-1	1,32	0,16	0,55	-1,93
XM_917709	**Krtap8-1**	keratin associated protein 8-1	1,54	NA	0,20	-1,84
NM_010676	**Krtap8-2**	keratin associated protein 8-2	2,05	0,39	0,12	-2,06
NM_029351	**Krtap9-3**	keratin associated protein 9-3	1,68	0,16	0,07	-1,79
NM_010685	**Lamp2**	lysosomal-associated membrane protein 2	0,33	-0,36	-1,24	-1,03
NM_024434	**Lap3**	leucine aminopeptidase 3	1,41	0,30	-0,06	-0,66
NM_025420	**Lce1m**	late cornified envelope 1M	-0,39	0,26	0,74	0,67
NM_008493	**Lep**	leptin	2,27	0,65	3,74	1,90
NM_144862	**Lims2**	LIM and senescent cell antigen like domains 2	NA	0,31	1,43	0,63
NM_029796	**Lrg1**	leucine-rich alpha-2-glycoprotein 1	0,99	1,06	1,51	0,88
XM_919092	**Lrrc17**	leucine rich repeat containing 17	-1,12	-0,68	-1,09	-0,81
NM_019391	**Lsp1**	lymphocyte specific 1	-0,65	-0,72	-0,26	-0,32
NM_008524	**Lum**	lumican	-0,90	-0,99	-1,44	-0,76
NM_013590	**Lyz1**	lysozyme 1	-0,51	-0,78	-0,53	-1,00
NM_145569	**Mat2a**	methionine adenosyltransferase II, alpha	0,44	0,05	-1,16	-1,25
NM_019648	**Metap2**	methionine aminopeptidase 2	0,77	-0,09	-0,83	-1,31
NM_029568	**Mfap4**	microfibrillar-associated protein 4	-1,10	-1,03	-0,59	-1,05
NM_146035	**Mgat2**	mannoside acetylglucosaminyltransferase 2	0,14	-0,32	-0,77	-0,62
NM_008597	**Mgp**	matrix Gla protein	-0,74	-0,76	-0,08	-0,89
NM_010809	**Mmp3**	matrix metallopeptidase 3	-0,72	-0,24	-1,51	-0,98
NM_024431	**Morf4l1**	mortality factor 4 like 1	-0,28	-0,48	-0,84	-0,85
NM_019768	**Morf4l2**	mortality factor 4 like 2	0,17	-0,25	-0,77	-0,67
NM_026835	**Ms4a6d**	membrane-spanning 4-domains, subfamily A, member 6D	0,69	0,61	NA	-0,23
NM_027836	**Ms4a7**	membrane-spanning 4-domains, subfamily A, member 7	NA	-0,48	-0,99	-0,70
NM_013602	**Mt1**	metallothionein 1	1,63	1,34	0,89	0,03
NM_008630	**Mt2**	metallothionein 2	1,78	1,24	0,24	-0,31
NM_008631	**Mt4**	metallothionein 4	1,18	0,34	0,30	-0,78
NM_010855	**Myh4**	myosin, heavy polypeptide 4, skeletal muscle	-2,29	-0,19	0,12	0,96
NM_021285	**Myl1**	myosin, light polypeptide 1	-0,85	-0,38	-1,19	-0,84
NM_025972	**Naaa**	N-acylethanolamine acid amidase	-0,71	-0,68	-0,95	-0,84
NM_180960	**Nnat**	neuronatin	0,32	0,68	1,97	2,20
NM_008714	**Notch1**	Notch gene homolog 1 (Drosophila)	0,65	1,01	0,97	NA
NM_010930	**Nov**	nephroblastoma overexpressed gene	-0,79	-0,80	NA	NA
NM_001003914	**Obscn**	obscurin, cytoskeletal calmodulin and titin-interacting RhoGEF	NA	0,66	NA	0,64
NM_008760	**Ogn**	osteoglycin	-0,40	-0,89	-0,62	-1,04
NM_011026	**P2rx4**	purinergic receptor P2X, ligand-gated ion channel 4	-0,61	-0,40	NA	-0,73
NM_008774	**Pabpc1**	poly(A) binding protein, cytoplasmic 1	0,61	0,22	-0,40	-0,68
NM_027995	**Paqr7**	progestin and adipoQ receptor family member VII	-0,60	-0,64	-0,61	0,41
NM_011044	**Pck1**	phosphoenolpyruvate carboxykinase 1, cytosolic	NA	0,57	2,25	0,79

NM_021514	**Pfkm**	phosphofructokinase, muscle	-0,66	0,38	0,48	0,63
NM_018870	**Pgam2**	phosphoglycerate mutase 2	-1,06	0,27	0,62	0,41
NM_013750	**Phlda3**	pleckstrin homology-like domain, family A, member 3	0,15	0,40	1,19	0,95
NM_177298	**Pisd**	phosphatidylserine decarboxylase	-1,05	-1,25	NA	-0,38
XM_001476056	**Pisd-ps2**	phosphatidylserine decarboxylase, pseudogene 2	-0,86	-0,72	NA	0,15
NM_145131	**Pitrm1**	pitrilysin metallepetidase 1	0,95	1,09	NA	NA
NM_139269	**Pla2g16**	phospholipase A2, group XVI	-0,39	0,12	1,07	0,71
NM_015784	**Postn**	periostin, osteoblast specific factor	NA	-0,92	-1,44	-1,66
NM_008247	**Ppap2a**	phosphatidic acid phosphatase type 2A	-0,63	-0,42	-1,08	-1,06
NM_009358	**Ppp2r5d**	protein phosphatase 2, regulatory subunit B (B56), delta isoform	NA	0,40	0,85	0,60
NM_011156	**Prep**	prolyl endopeptidase	0,65	0,60	-0,09	-0,04
NM_011171	**Procr**	protein C receptor, endothelial	1,25	0,86	0,59	-0,19
NM_175249	**Psapl1**	prosaposin-like 1	-0,36	-0,80	-0,93	-0,29
NM_011965	**Psma1**	proteasome (prosome, macropain) subunit, alpha type 1	0,18	-0,18	-0,89	-0,69
NM_020576	**Psors1c2**	psoriasis susceptibility 1 candidate 2 (human)	1,61	0,44	0,23	-1,63
NM_008973	**Ptn**	pleiotrophin	-0,69	-0,78	NA	-0,66
NM_008975	**Ptp4a3**	protein tyrosine phosphatase 4a3	-0,30	0,52	1,34	1,47
NM_181070	**Rab18**	RAB18, member RAS oncogene family	0,05	-0,30	-0,83	-0,68
NM_031874	**Rab3d**	RAB3D, member RAS oncogene family	-0,34	0,45	0,93	0,62
NM_011239	**Ranbp1**	RAN binding protein 1	0,51	-0,08	-0,76	-0,81
XM_001475793	**Rarres1**	retinoic acid receptor responder (tazarotene induced) 1	NA	-0,58	-1,19	-0,76
NM_027852	**Rarres2**	retinoic acid receptor responder (tazarotene induced) 2	-0,50	0,14	0,88	0,82
NM_025936	**Rars**	arginyl-tRNA synthetase	0,71	0,05	NA	-0,80
NM_016809	**Rbm3**	RNA binding motif protein 3	-0,09	-0,11	-0,97	-0,74
NM_020509	**Retnla**	resistin like alpha	-0,69	-0,26	0,99	-0,34
NM_023275	**Rhoj**	ras homolog gene family, member J	0,08	-0,21	-0,60	-0,73
NM_145491	**Rhoq**	ras homolog gene family, member Q	NA	0,02	0,63	0,60
XM_911372	**Ribc2**	RIB43A domain with coiled-coils 2	1,68	0,40	0,48	-1,85
NM_001033135	**Rnf149**	ring finger protein 149	0,89	0,21	-0,33	-0,76
NM_007475	**Rplp0**	ribosomal protein, large, P0	0,05	-0,30	-0,66	-0,85
NM_011310	**S100a3**	S100 calcium binding protein A3	1,56	0,82	0,44	-1,59
NM_013650	**S100a8**	S100 calcium binding protein A8 (calgranulin A)	2,16	2,85	-0,69	-0,58
NM_009114	**S100a9**	S100 calcium binding protein A9 (calgranulin B)	2,69	2,93	-0,85	-0,37
NM_011315	**Saa3**	serum amyloid A 3	1,12	1,11	NA	NA
NM_009127	**Scd1**	stearoyl-Coenzyme A desaturase 1	-0,62	-0,35	0,74	0,25
NM_026563	**Sdccag3**	serologically defined colon cancer antigen 3	-1,18	-0,64	NA	NA
NM_172702	**Serinc2**	serine incorporator 2	0,91	0,63	NA	NA
NM_173024	**Serpina3b**	serine (or cysteine) peptidase inhibitor, clade A, member 3B	-0,68	-1,03	-1,25	-0,28
NM_009252	**Serpina3n**	serine (or cysteine) peptidase inhibitor, clade A, member 3N	0,79	0,85	0,43	0,36
NM_025429	**Serpinb1a**	serine (or cysteine) peptidase inhibitor, clade B, member 1a	-0,30	0,13	-1,09	-0,63

NM_008871	**Serpine1**	serine (or cysteine) peptidase inhibitor, clade E, member 1	1,42	2,11	NA	NA
NM_018754	**Sfn**	stratifin	0,78	0,06	-0,70	-0,94
NM_009144	**Sfrp2**	secreted frizzled-related protein 2	-1,37	-1,55	-0,83	-1,83
NM_146083	**Sfrs7**	splicing factor, arginine/serine-rich 7	0,40	-0,21	-1,08	-0,66
NM_021882	**Si**	silver	2,35	1,31	0,51	NA
NM_011378	**Sin3a**	transcriptional regulator, SIN3A (yeast)	-0,14	0,71	0,31	0,64
XM_916404	**Skint10**	selection and upkeep of intraepithelial T cells 10	-1,05	-0,25	0,67	0,70
NM_029612	**Slamf9**	SLAM family member 9	NA	-0,61	-0,66	NA
NM_009201	**Slc1a5**	solute carrier family 1 (neutral amino acid transporter), member 5	0,04	0,48	1,20	0,67
NM_027872	**Slc46a3**	solute carrier family 46, member 3	-0,72	-0,63	-0,32	-0,45
NM_175316	**Slco2b1**	solute carrier organic anion transporter family, member 2b1	-1,46	-0,60	0,01	0,08
NM_011410	**Slfn4**	schlafen 4	2,37	1,05	NA	NA
NM_025357	**Smpx**	small muscle protein, X-linked	-1,07	-0,28	-0,73	-0,70
NM_011430	**Sncg**	synuclein, gamma	NA	0,05	1,73	0,97
NM_009166	**Sorbs1**	sorbin and SH3 domain containing 1	-0,24	0,40	0,96	0,75
NM_007463	**Speg**	SPEG complex locus	0,59	-0,10	1,00	-1,30
NM_011471	**Sprr2e**	small proline-rich protein 2E	-0,18	-0,04	-0,73	-0,68
NM_011157	**Srgn**	serglycin	1,21	0,85	NA	NA
NM_009183	**St8sia4**	ST8 alpha-N-acetyl-neuraminide alpha-2,8-sialyltransferase 4	-0,97	-0,75	-0,05	-0,17
NM_001082543	**Stfa1**	stefin A1	0,45	0,90	-1,88	0,83
NM_194334	**Tbc1d2b**	TBC1 domain family, member 2B	-0,61	0,10	0,00	0,59
XM_001474960	**Tceal7**	transcription elongation factor A (SII)-like 7	-1,59	-1,31	-1,69	-1,51
XM_904350	**Tchh**	trichohyalin	1,17	0,12	-0,88	-2,78
NM_027762	**Tchhl1**	trichohyalin-like 1	1,10	-0,10	-0,53	-2,05
NM_008536	**Tm4sf1**	transmembrane 4 superfamily member 1	0,68	0,11	-0,23	-0,97
NM_023056	**Tmem176b**	transmembrane protein 176B	-0,54	-0,73	-0,53	-0,62
NM_019631	**Tmem45a**	transmembrane protein 45a	-0,60	-0,81	-1,01	-0,89
NM_009400	**Tnfrsf18**	tumor necrosis factor receptor superfamily, member 18	-0,01	-0,05	0,84	0,86
NM_022322	**Tnmd**	tenomodulin	-0,58	-0,62	-0,80	-0,45
NM_009405	**Tnni2**	troponin I, skeletal, fast 2	-1,12	0,27	0,44	0,61
NM_011620	**Tnnt3**	troponin T3, skeletal, fast	-1,15	-0,84	-0,16	-0,25
NM_133977	**Trf**	transferrin	-0,01	0,30	1,52	0,76
NM_023655	**Trim29**	tripartite motif-containing 29	0,40	-0,27	-0,85	-0,63
NM_025863	**Trim59**	tripartite motif-containing 59	0,75	-0,03	NA	-0,83
NM_146010	**Tspan8**	tetraspanin 8	-0,40	0,12	1,04	0,96
NM_028004	**Ttn**	titin	-1,28	-0,45	NA	-0,94
NM_009450	**Tubb2a**	tubulin, beta 2A	0,76	0,12	-0,62	-0,45
XM_001479032	**Tubb2c-ps1**	tubulin, beta 2c, pseudogene 1	0,45	-0,84	-0,86	-0,73
NM_001042523	**Txnrd1**	thioredoxin reductase 1	0,63	0,62	0,37	0,24
NM_025356	**Ube2d3**	ubiquitin-conjugating enzyme E2D 3 (UBC4/5 homolog, yeast)	0,25	-0,20	-0,79	-0,93

NM_009466	**Ugdh**	UDP-glucose dehydrogenase	0,79	0,65	NA	NA
NM_201645	**Ugt1a1**	UDP glucuronosyltransferase 1 family, polypeptide A1	-0,10	-0,77	-0,65	NA
NM_019449	**Unc93b1**	unc-93 homolog B1 (C. elegans)	-0,67	-0,28	-0,25	-0,62
NM_134174	**V1rc19**	vomeronasal 1 receptor, C19	1,48	0,57	-0,08	-1,55
NM_177723	**Vsig8**	V-set and immunoglobulin domain containing 8	1,53	0,84	0,21	-0,61
NM_172767	**Vwa5a**	von Willebrand factor A domain containing 5A	0,97	0,99	NA	NA
NM_172677	**Ythdf3**	YTH domain family 3	-0,28	-0,37	-0,88	-0,73
XR_034420	**Zfp534**	zinc finger protein 534	NA	-0,82	NA	-0,74
NM_009517	**Zmat3**	zinc finger matrin type 3	-0,11	-0,13	0,98	0,73
XM_001472815			-0,73	-1,08	-1,40	-1,85
XR_031876			1,63	0,51	0,32	-1,72
NM_010821			-0,83	-0,93	-1,25	-1,30
XM_001477703			0,39	-0,91	-1,74	-1,24
XM_913492			0,18	-0,16	-1,13	-1,17
XR_031479			NA	-0,21	-0,98	-1,10
XR_032297			0,64	-0,52	-1,15	-1,07
XR_033075			0,61	-0,08	-0,87	-0,93
XM_983502			-0,45	-0,33	-0,97	-0,92
XM_001473443			-0,75	-0,39	-0,83	-0,91
XM_001473340			1,80	1,26	0,73	-0,87
XM_925747			0,38	-0,10	-1,07	-0,86
XR_032886			-0,50	-0,41	-0,80	-0,85
XM_001473623			-0,49	-0,34	-0,84	-0,83
XM_001471641			-0,61	-0,69	-0,85	-0,81
XR_033355			-0,45	-0,26	-0,84	-0,78
NM_025518			1,44	0,98	0,45	-0,77
XR_004622			0,30	-0,20	-0,79	-0,73
XM_001476723			-0,35	-0,31	-0,84	-0,71
XR_034668			0,08	-0,37	-0,99	-0,70
NM_001080710			-0,39	-0,73	-1,32	-0,69
NM_026637			0,02	-0,03	-0,70	-0,62
XR_034014			-0,40	-0,24	-0,72	-0,60
XR_034372			-0,08	-0,70	NA	-0,59
XR_031213			0,65	-0,07	-0,56	-0,59
XM_980395			1,36	0,69	0,35	-0,49
XM_001475422			0,68	0,47	-0,86	-0,49
NM_029821			0,79	0,83	0,09	0,33
NM_177669			-0,75	-0,14	0,66	0,57
XM_001479087			0,35	0,62	0,38	0,73
XM_001478015			0,14	0,66	NA	0,74

XM_001477665	NA	-0,11	0,62	0,77
XM_001471690	2,06	0,76	0,48	NA
NM_001081087	-1,15	-1,10	NA	NA
XM_001475886	1,74	0,78	NA	NA
