# Supplementary material for: Dose-Dependent Onset of Regenerative Program in Neutron Irradiated Mouse Skin
Source: PLoS One. 2011 Apr 27;6(4):e19242. doi: 10.1371/journal.pone.0019242 (PMC3083422; doi:10.1371/journal.pone.0019242)
Supplement: Table S2 — Functional Annotation Clustering of modulated genes. Functional Annotation Clustering report of 440 genes modulated at least 1.5 fold in two different conditions in comparison to the sham-irradiated control using DAVID (see text, Ref. 49) and (http://david.abcc.ncifcrf.gov/home.jsp) web tool. The grouping algorithm is based on the hypothesis that similar annotations should have similar gene members. (PDF) [file pone.0019242.s004.pdf]

TABLE S2. Gene Ontology analysis of modulated genes.

Term	Count	%	PValue	List Total	Pop Hits	Pop Total	Fold Enrichment	Bonferroni	Benjamini	FDR
Keratin	32	10,53%	3,87E-34	258	87	16241	23,15	3,35E-31	3,35E-31	6,00E-31
GO:0005882~intermediate filament	34	11,18%	1,47E-31	258	121	15845	17,26	1,15E-28	1,15E-28	2,25E-28
GO:0045111~intermediate filament cytoskeleton	34	11,18%	3,60E-31	258	124	15845	16,84	2,82E-28	1,41E-28	5,51E-28
GO:0044430~cytoskeletal part	47	15,46%	3,18E-19	258	589	15845	4,90	2,49E-16	8,31E-17	4,87E-16
GO:0005200~structural constituent of cytoskeleton	18	5,92%	1,27E-14	232	93	16377	13,66	3,45E-11	3,45E-11	2,27E-11
intermediate filament	17	5,59%	1,42E-14	258	71	16241	15,07	1,23E-11	6,16E-12	2,20E-11
mmu01430:Cell Communication	24	7,89%	1,22E-13	109	131	4079	6,86	2,39E-11	2,39E-11	1,53E-10
IPR001664:Intermediate filament protein	16	5,26%	2,07E-13	256	73	16924	14,49	1,22E-09	1,22E-09	4,01E-10
PIRSF002282:cytoskeletal keratin	15	4,93%	1,86E-12	168	49	7368	13,43	5,82E-09	5,82E-09	3,36E-09
GO:0005856~cytoskeleton	48	15,79%	6,74E-12	258	957	15845	3,08	5,28E-09	1,32E-09	1,03E-08
GO:0005198~structural molecule activity	32	10,53%	1,79E-09	232	638	16377	3,54	4,84E-06	2,42E-06	3,19E-06
GO:0007010~cytoskeleton organization and biogenesis	26	8,55%	3,53E-07	221	537	14977	3,28	1,83E-03	1,83E-03	6,73E-04
GO:0043228~non-membrane-bound organelle	52	17,11%	2,30E-06	258	1614	15845	1,98	1,80E-03	2,57E-04	3,52E-03
GO:0043232~intracellular non-membrane-bound organelle	52	17,11%	2,30E-06	258	1614	15845	1,98	1,80E-03	2,57E-04	3,52E-03
IPR003054:Type II keratin	6	1,97%	4,60E-05	256	27	16924	14,69	2,36E-01	8,60E-02	8,89E-02
GO:0044446~intracellular organelle part	67	22,04%	1,01E-04	258	2610	15845	1,58	7,62E-02	5,27E-03	1,55E-01
GO:0044422~organelle part	67	22,04%	1,15E-04	258	2621	15845	1,57	8,60E-02	5,61E-03	1,76E-01
GO:0006996~organelle organization and biogenesis	33	10,86%	0,001247	221	1241	14977	1,80	9,98E-01	2,89E-01	2,36E+00
GO:0016043~cellular component organization and biogenesis	50	16,45%	0,057505	221	2686	14977	1,26	1,00E+00	9,91E-01	6,77E+01
Coiled coil	24	7,89%	0,300055	258	1286	16241	1,17	1,00E+00	9,96E-01	9,96E+01

Term	Count	%	PValue	List Total	Pop Hits	Pop Total	Fold Enrichment	Bonferroni	Benjamini	FDR
GO:0005200~structural constituent of cytoskeleton	18	5,92%	0	232	93	16377	13,66	3,45E-11	3,45E-11	2,27E-11
intermediate filament	17	5,59%	0	258	71	16241	15,07	1,23E-11	6,16E-12	2,20E-11
mmu01430:Cell Communication	24	7,89%	0	109	131	4079	6,86	2,39E-11	2,39E-11	1,53E-10
IPR001664:Intermediate filament protein	16	5,26%	0	256	73	16924	14,49	1,22E-09	1,22E-09	4,01E-10
PIRSF002282:cytoskeletal keratin	15	4,93%	0	168	49	7368	13,43	5,82E-09	5,82E-09	3,36E-09
IPR002957:Keratin, type I	12	3,95%	0	256	40	16924	19,83	6,76E-08	3,38E-08	2,23E-08
region of interest:Coil 2	9	2,96%	2,33E-008	208	24	9547	17,21	3,42E-04	3,42E-04	4,94E-05
region of interest:Linker 12	9	2,96%	6,65E-008	208	27	9547	15,30	9,77E-04	4,88E-04	1,41E-04
region of interest:Coil 1B	9	2,96%	1,24E-007	208	29	9547	14,24	1,82E-03	4,55E-04	2,62E-04
region of interest:Coil 1A	9	2,96%	1,24E-007	208	29	9547	14,24	1,82E-03	4,55E-04	2,62E-04

region of interest:Linker 1	9	2,96%	1,24E-007	208	29	9547	14,24	1,82E-03	4,55E-04	2,62E-04
region of interest:Rod	9	2,96%	1,66E-007	208	30	9547	13,77	2,43E-03	4,06E-04	3,51E-04
region of interest:Tail	9	2,96%	2,19E-007	208	31	9547	13,33	3,22E-03	4,60E-04	4,65E-04
site:Stutter	7	2,30%	6,46E-007	208	16	9547	20,08	9,45E-03	1,19E-03	1,37E-03
region of interest:Head	8	2,63%	2,71E-006	208	30	9547	12,24	3,91E-02	4,42E-03	5,75E-03
Coiled coil	24	7,89%	0,300055	258	1286	16241	1,17	1,00E+00	9,96E-01	9,96E+01

Term	Count	%	PValue	List Total	Pop Hits	Pop Total	Fold Enrichment	Bonferroni	Benjamini	FDR
Secreted	45	14,80%	1,29E-007	258	1200	16241	2,36	1,12E-04	3,73E-05	2,00E-04
signal	73	24,01%	3,16E-007	258	2551	16241	1,80	2,74E-04	5,48E-05	4,91E-04
GO:0044421~extracellular region part	63	20,72%	6,15E-006	258	2195	15845	1,76	4,81E-03	4,02E-04	9,41E-03
GO:0005576~extracellular region	65	21,38%	1,92E-005	258	2375	15845	1,68	1,49E-02	1,16E-03	2,94E-02
GO:0005615~extracellular space	58	19,08%	3,15E-005	258	2064	15845	1,73	2,44E-02	1,76E-03	4,82E-02
signal peptide	67	22,04%	0,000432	208	2081	9547	1,48	9,98E-01	4,11E-01	9,12E-01

Term	Count	%	PValue	List Total	Pop Hits	Pop Total	Fold Enrichment	Bonferroni	Benjamini	FDR
immune response	14	4,61%	6,11E-007	258	147	16241	6,00	5,29E-04	6,62E-05	9,47E-04
GO:0006955~immune response	25	8,22%	2,18E-006	221	554	14977	3,06	1,12E-02	5,64E-03	4,16E-03
GO:0009605~response to external stimulus	22	7,24%	1,37E-005	221	496	14977	3,01	6,88E-02	1,77E-02	2,62E-02
GO:0002376~immune system process	29	9,54%	6,21E-005	221	859	14977	2,29	2,76E-01	3,52E-02	1,19E-01
GO:0009611~response to wounding	16	5,26%	7,39E-005	221	318	14977	3,41	3,19E-01	3,76E-02	1,41E-01
GO:0006954~inflammatory response	13	4,28%	0,000137	221	227	14977	3,88	5,09E-01	6,26E-02	2,61E-01
GO:0006952~defense response	22	7,24%	0,002468	221	728	14977	2,05	1,00E+00	4,14E-01	4,61E+00
GO:0006950~response to stress	23	7,57%	0,006086	221	839	14977	1,86	1,00E+00	6,40E-01	1,10E+01
GO:0050896~response to stimulus	57	18,75%	0,209015	221	3439	14977	1,12	1,00E+00	1,00E+00	9,89E+01

Term	Count	%	PValue	List Total	Pop Hits	Pop Total	Fold Enrichment	Bonferroni	Benjamini	FDR
GO:0030017~sarcomere	10	3,29%	1,64E-006	258	69	15845	8,90	1,28E-03	2,14E-04	2,51E-03
GO:0044449~contractile fiber part	10	3,29%	2,36E-006	258	72	15845	8,53	1,85E-03	2,05E-04	3,61E-03
GO:0030016~myofibril	10	3,29%	4,17E-006	258	77	15845	7,98	3,26E-03	3,27E-04	6,38E-03
GO:0043292~contractile fiber	10	3,29%	5,74E-006	258	80	15845	7,68	4,49E-03	4,09E-04	8,79E-03
GO:0003012~muscle system process	9	2,96%	6,16E-005	221	92	14977	6,63	2,74E-01	3,92E-02	1,18E-01
GO:0006936~muscle contraction	9	2,96%	6,16E-005	221	92	14977	6,63	2,74E-01	3,92E-02	1,18E-01

GO:0031674~I band	6	1,97%	0,000235	258	35	15845	10,53	1,69E-01	1,08E-02	3,60E-01
Muscle protein	6	1,97%	0,000626	258	44	16241	8,58	4,19E-01	4,82E-02	9,67E-01
GO:0006937~regulation of muscle contraction	4	1,32%	0,015631	221	36	14977	7,53	1,00E+00	8,51E-01	2,60E+01
GO:0015629~actin cytoskeleton	9	2,96%	0,027361	258	220	15845	2,51	1,00E+00	5,40E-01	3,46E+01
GO:0003008~system process	15	4,93%	0,999259	221	1894	14977	0,54	1,00E+00	1,00E+00	1,00E+02

Term	Count	%	PValue	List Total	Pop Hits	Pop Total	Fold Enrichment	Bonferroni	Benjamini	FDR
signal	73	24,01%	3,16E-007	258	2551	16241	1,80	2,74E-04	5,48E-05	4,91E-04
glycoprotein	77	25,33%	1,05E-005	258	3012	16241	1,61	9,03E-03	1,01E-03	1,62E-02
signal peptide	67	22,04%	0,000432	208	2081	9547	1,48	9,98E-01	4,11E-01	9,12E-01
disulfide bond	58	19,08%	0,000449	208	1725	9547	1,54	9,99E-01	3,98E-01	9,47E-01
glycosylation site:N-linked (GlcNAc...)	68	22,37%	0,005136	208	2325	9547	1,34	1,00E+00	9,85E-01	1,03E+01
transmembrane region	52	17,11%	0,571673	208	2384	9547	1,00	1,00E+00	1,00E+00	1,00E+02

Term	Count	%	PValue	List Total	Pop Hits	Pop Total	Fold Enrichment	Bonferroni	Benjamini	FDR
GO:0009605~response to external stimulus	22	7,24%	1,37E-005	221	496	14977	3,01	6,88E-02	1,77E-02	2,62E-02
GO:0006935~chemotaxis	10	3,29%	4,30E-005	221	113	14977	6,00	2,00E-01	4,37E-02	8,22E-02
GO:0042330~taxis	10	3,29%	4,30E-005	221	113	14977	6,00	2,00E-01	4,37E-02	8,22E-02
GO:0007610~behavior	15	4,93%	0,000488	221	338	14977	3,01	9,21E-01	1,55E-01	9,28E-01
GO:0007626~locomotory behavior	11	3,62%	0,001508	221	219	14977	3,40	1,00E+00	3,24E-01	2,84E+00
cytokine	10	3,29%	0,001695	258	172	16241	3,66	7,70E-01	1,15E-01	2,60E+00
inflammation	4	1,32%	0,001883	258	16	16241	15,74	8,05E-01	1,18E-01	2,88E+00
chemotaxis	6	1,97%	0,001888	258	56	16241	6,74	8,06E-01	1,10E-01	2,89E+00
GO:0008009~chemokine activity	5	1,64%	0,002154	232	39	16377	9,05	9,97E-01	8,57E-01	3,76E+00
GO:0042379~chemokine receptor binding	5	1,64%	0,002367	232	40	16377	8,82	9,98E-01	7,99E-01	4,13E+00
inflammatory response	6	1,97%	0,004421	258	68	16241	5,55	9,79E-01	2,02E-01	6,64E+00
GO:0042221~response to chemical stimulus	16	5,26%	0,008207	221	508	14977	2,13	1,00E+00	6,57E-01	1,46E+01
GO:0001664~G-protein-coupled receptor binding	5	1,64%	0,012663	232	64	16377	5,51	1,00E+00	9,97E-01	2,03E+01
SM00199:SCY	4	1,32%	0,013861	125	37	9059	7,83	1,00E+00	1,00E+00	1,87E+01
GO:0005102~receptor binding	18	5,92%	0,015664	232	677	16377	1,88	1,00E+00	9,98E-01	2,45E+01
IPR001811:Small chemokine, interleukin-8-like	4	1,32%	0,018015	256	37	16924	7,15	1,00E+00	1,00E+00	2,97E+01
IPR000827:Small chemokine, C-C	3	0,99%	0,039344	256	21	16924	9,44	1,00E+00	1,00E+00	5,40E+01
GO:0005125~cytokine activity	8	2,63%	0,045835	232	231	16377	2,44	1,00E+00	1,00E+00	5,66E+01
PIRSF001950:small inducible chemokine, C/CC types	3	0,99%	0,074329	168	20	7368	6,58	1,00E+00	1,00E+00	7,53E+01
mmu04060:Cytokine-cytokine receptor interaction	11	3,62%	0,105313	109	242	4079	1,70	1,00E+00	9,55E-01	7,51E+01
