# Supplementary material for: Dose-Dependent Onset of Regenerative Program in Neutron Irradiated Mouse Skin
Source: PLoS One. 2011 Apr 27;6(4):e19242. doi: 10.1371/journal.pone.0019242 (PMC3083422; doi:10.1371/journal.pone.0019242)
Supplement: Table S3 — Immunohistochemistry imaging analysis. Immunoreactivities expresssed as arbitrary densitometric units 95% confidential interval min-max median value. After normalization for the intensity of background staining, at least four areas for each slide were scored by ImageJ dedicated software for covering of the epidermal layer and hair follicles. (PDF) [file pone.0019242.s005.pdf]

Table S3. Arbitrary densitometric units. 95% Confidential interval min-max median value

	Untreated		0.2 Gy-6 H.		0.2 Gy-24 H.		1 Gy-6 H.		1 Gy-24H.	
	**Min**	**Max**	**Min**	**Max**	**Min**	**Max**	**Min**	**Max**	**Min**	**Max**
**Krt-6**	47.1	87.4	91.2	119	103.1	110.9	32.3	92	44.5	91
**Krt-10**	53.0	83.7	47.8	78.6	49.4	97.1	68.9	78.2	52.6	81.2
**S100A-8**	12.2	29.6	42.2	60.1	49.1	73.3	8.2	35.0	13.2	21.5
**S100A-9**	12.4	22.3	39.2	62.5	23.2	71.7	13.9	43.8	18.6	41.0
**Cas-3**	12.6	14.2	15.8	34.0	14.4	30.3	30.1	39.1	34.1	45.1
**TUNEL**	3.3	9.6	4.2	12.7	0.4	7.3	11.3	19.1	7.8	12.2
**8-oxo-G**	5.3	10.0	36.9	46.5	3.8	9.4	54.2	58.3	34.1	45.1
