# Supplementary material for: Dose-Dependent Onset of Regenerative Program in Neutron Irradiated Mouse Skin
Source: PLoS One. 2011 Apr 27;6(4):e19242. doi: 10.1371/journal.pone.0019242 (PMC3083422; doi:10.1371/journal.pone.0019242)
Supplement: Appendix S1 — Algorithms for neutron microdosimetry. Multiple ionization event numbers (n ) for different site sizes and the absorbed dose (D ) values calculated by using microdosimetric algorithms (see Fig. S1 and Ref. 45, 46). (DOC) [file pone.0019242.s006.doc]

**Appendix**

Microdosimetry is a conceptual framework for the analysis of microscopic distribution of energy in irradiated matter. It has developed both theoretical and experimental tools to investigate the absorbed energy distributions in sites of any size. When neutrons irradiate living tissues, mammalian cells experience ionization and excitation events due to charged particle set in motion by neutrons. The total amount ***ε*** of absorbed energy in a given site, due to the passage of a single ionizing particle through or close to the site, depends on the site size, particle velocity and electrical charge. The absorbed energy divided by site mean chord $\bar{l}$ is called *lineal energy*: ***y =*** ***ε/*** $\bar{l}$. The absorbed energy divided by site mass ***m*** is called *specific energy*: ***z*** = ***ε/ m***. At a given dose ***D***, the absorbed energy in a given site can be due to one (single event) or more (multiple events) ionizing particles. The average event number is , where $\bar{z}_{F}$ is the average value of ***z***. The probability that exactly ***n*** events occur in the site, assuming that they follow Poisson statistics, is: . If the site is spherical, we can easily substitute ***z*** with ***y***. Then , Where $\bar{y}_{F}$ is the frequency mean value of ***y***, in keV/µm, ***d*** is the site diameter in µm, ***D*** in Gray.

The probability ***F_2_*** that a given site experiences 2 or more independent events is called cumulative probability. It is straightforward to write ***F2 = 1-P(0)-P(1)***, where ***P(0)*** and ***P(1)*** are the probabilities to have in the site 0 or 1 event respectively. Therefore, substituting ***P(0)*** and ***P(1)*** with their explicit expressions, we obtain:

.
