# Supplementary material for: Dose-Dependent Onset of Regenerative Program in Neutron Irradiated Mouse Skin
Source: PLoS One. 2011 Apr 27;6(4):e19242. doi: 10.1371/journal.pone.0019242 (PMC3083422; doi:10.1371/journal.pone.0019242)
Supplement: Appendix S2 — Radiobiological units and abbreviations used in the text. (DOC) [file pone.0019242.s007.doc]

**Radiobiological units and abbreviations used in the text**

**Gy** The gray is the unit of absorbed radiation dose of ionizing radiation and is defined as the absorption of one joule of ionizing radiation by one kilogram of matter

**Sv** The sievert is the derived unit of dose equivalent radiation. It attempts to quantitatively evaluate the biological effects of ionizing radiation as opposed to the physical aspects, which are characterised by the absorbed dose, measured in Gy.

**RBE** Relative Biological Effectiveness is traditionally defined as the ratio of a dose of a standard

low linear energy transfer X ray beam (x ray of 250KeV energy) (*DX*) to the dose of the test radiation type or configuration (*DT*), required to cause the same biological level of effect. RBE is defined by the formula *DX*/ *DT*

**LET** The average amount of energy that is lost over a defined distance; for example the energy deposited in ten cells is known as the Linear Energy Transfer (LET).

**MeV** It is a common unit of energy within physics. 1 MeV equals 1.602×10^−13^
 Joule, about twice the rest mass-energy of an electron.

**ROS** Reactive oxygen species (ROS) are chemically-reactive molecules containing oxygen,

including oxygen ions and peroxides. ROS are highly reactive due to the presence of unpaired valence shell electrons.
